# Stabilizing Proteins by Chemical Cross-Linking: Insights into Conformation, Unfolding, and Aggregation Using Native Ion Mobility Mass Spectrometry

**DOI:** 10.1021/acs.analchem.5c06844

**Published:** 2025-11-17

**Authors:** Raya Sadighi, Rosalin M. A. van Paasen, George H. Hutchins, Ivar D. Jansen, Saskia Neubacher, Tom N. Grossmann, Anouk M. Rijs

**Affiliations:** † Division of Bioanalytical Chemistry (MS-Laserlab), Department of Chemistry and Pharmaceutical Sciences, Amsterdam Institute of Molecular and Life Sciences, 1190 Vrije Universiteit Amsterdam, De Boelelaan 1085, 1081 HV Amsterdam, The Netherlands; ‡ Centre for Analytical Sciences Amsterdam (CASA), The Netherlands; § Incircular B.V., De Boelelaan 1085, 1081 HZ Amsterdam, The Netherlands; ∥ Department of Chemistry and Pharmaceutical Sciences, Amsterdam Institute of Molecular and Life Sciences, 1190 Vrije Universiteit Amsterdam, De Boelelaan 1085, 1081 HZ Amsterdam, The Netherlands; ⊥ Institute of Organic and Biomolecular Chemistry, Faculty of Chemistry, Georg-August-Universität Göttingen, Tammanstr. 2, 37077 Göttingen, Germany

## Abstract

The function and stability of proteins depend on their three-dimensional structure, which includes conformational dynamics and potential self-assembly. Protein structural organization is particularly important in biotechnological applications, where protein integrity is often challenged by nonphysiological conditions, leading to disassembly, aggregation, and eventually the loss of function or activity. The use of chemical cross-linking strategies, such as the in situ cyclization of proteins (INCYPRO), can overcome these challenges, providing proteins and protein complexes with enhanced resistance to thermal and chemical stress. To probe how cross-linking affects protein structure and stability, we combined native ion mobility mass spectrometry (nIM-MS) and collision-induced unfolding (CIU). Here, we compare the wild-type (WT) and chemically cross-linked trimeric complex of *Pseudomonas fluorescens* Esterase (PFE), using nIM-MS to obtain high-resolution insights into the conformations, while CIU allowed the investigation of unfolding pathways and structural resilience under activation. We show that upon gas-phase activation, the native enzyme undergoes extensive unfolding and dissociation into monomers, whereas the cross-linked form remains compact and structurally intact. CIU fingerprints show that, when combining tunnel-in pressure and DC voltages, WT-PFE undergoes extensive structural unfolding, whereas the cross-linked complex resists conformational transitions. This mass spectrometry platform offers a powerful approach to study protein stability and, in this case, highlights the potential of protein cross-linking for preserving native-like structures.

## Graphical abstract

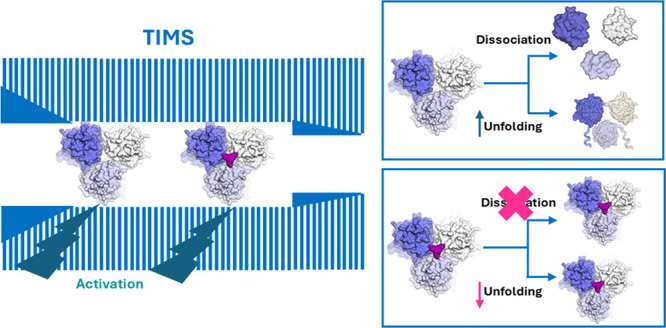

## Introduction

Proteins are highly adaptable molecular frameworks, whose biological functions depend on their native three-dimensional structure and ability to self-assemble into higher-order structures (HOS).^1^ These structural properties are essential for function, and even subtle changes, such as conformational shifts or partial unfolding, can promote aggregation and disrupt activity.^2–4^ The stabilization of protein quaternary structure, i.e. complex assemblies, remains a significant challenge due to the complexity of the interactions involved. Covalent cross-linking strategies offer a means to improve structural integrity of protein assemblies.^5,6^ To understand and optimize these stabilization approaches, robust methods to probe and characterize protein structure across different levels of organization are essential. The characterization of higher-order protein structures is often performed using X-ray crystallography providing atomic-level resolution,^7,8^ however, with limitations when analyzing proteins with multiple and dynamic conformations. Cryo-electron microscopy (cryo-EM) can preserve proteins in distinct hydrated states and is particularly effective for visualizing large complexes.^9,10^ However, both techniques face challenges in probing highly flexible regions and capturing the full extent of protein heterogeneity in particular under thermal or chemical stress.^11^ This is in principle possible with nuclear magnetic resonance (NMR) spectroscopy which allows the study of proteins in solution, offering unique insights into conformational flexibility. NMR spectroscopy provides valuable insights into protein flexibility, interaction, and dynamics. Although NMR is most powerful for smaller proteins, advanced methods such as TROSY and isotope labeling enable studies of very large (>MDa) assemblies, but these are highly specialized, nonroutine experiments that are technically demanding and resource-intensive.^12,13^ Other spectroscopic methods, such as dynamic light scattering (DLS), circular dichroism (CD),^14,15^ infrared spectroscopy (IR),^16,17^ and Raman spectroscopy^18,19^ can be used for protein analysis, however, their relatively low resolution hampers a detailed characterization of large protein complexes regarding their disassembly and aggregation behavior.

Native mass spectrometry (nMS) enables the analysis of intact proteins and protein complexes while preserving their native state in the gas phase.^20–22^ The nMS approach has emerged as a powerful technique in structural biology, offering an effective compromise between throughput and spatial resolution for the analysis of protein higher-order structures.^23^ An advantage of nMS is its ability to probe multiple conformations and proteoforms of protein complexes while using minimal sample amounts.^24,25^ However, the inherent complexity and heterogeneity of protein samples can lead to congested spectra, making data interpretation challenging.^26^ To improve the analysis of complex protein structures using nMS, separation techniques that maintain native conditions can be hyphenated with mass spectrometry. These include liquid chromatographic (LC) methods, such as size exclusion (SEC),^27,28^ ion exchange (IEX)^29,30^ and hydrophobic interaction chromatography (HIC).^31^ In addition, techniques such as capillary electrophoresis (CE)^32^ and field-flow-fractionation (FFF)^33^ can be employed. Among these techniques, SEC is commonly used to study stable protein aggregation due to its ability to separate molecular clusters based on their size. It is frequently employed in nMS workflows, where it functions as an effective online desalting step during the analysis native proteins.

nMS combined with ion mobility spectrometry (IMS) adds an extra analytical dimension via the gas-phase separation of biomolecules based on their mass, charge, size, and shapes.^25^ Several IM-MS methods and instruments are available, each with unique advantages for the analysis of protein complexes, which have been discussed in detail previously.^24^ Among these IM-MS methods, drift tube IMS (DTIM), traveling wave IMS (TWIMS), and trapped ion mobility spectrometry (TIMS) are commonly used for the study of the structure of protein complexes.^34–36^ TIMS has recently been employed for the characterization of native protein complexes. For example, Panczyk et al.^37^ used TIMS for analyzing large protein complexes in nMS, while the group of Bleiholder utilized a modified tandem-TIMS-Q-TOF to study glycoprotein structures and small proteins.^38,39^ Recently, TIMS has also been employed for the study of larger assemblies, including protein–DNA complexes.^40^ This application was demonstrated by Lin et al., who modified a commercial TIMS by changing the TIMS geometry and lowering the RF frequency in order to probe large biomolecular structures.^41^ Despite these advances, using nMS to study physiologically relevant structures in dynamic proteins still poses significant challenges. The use of higher transmission voltages and extended experimental time scales can induce structural changes, particularly when analyzing large protein complexes around 100 kDa.^3^

The use of covalently bound cross-linkers (CXL) has emerged as a powerful tool to stabilize the structure of large multiprotein complexes in nMS.^42^ This approach has been mainly focused on intermolecular disulfide bridges, noncanonical amino acids, and fusion proteins to covalently link monomeric units.^43–47^ Alternatively, the in situ cyclization of proteins (INCYPRO) was used to stabilize protein tertiary and quaternary structures.^48,49^ This approach utilizes C3-symmetric cross-linkers that react with three spatially aligned cysteine side chains to provide multicyclic protein topologies with a reduced unfolding tendency. For example, INCYPRO proved useful to stabilize the homotrimeric complex of *Pseudomonas fluorescens* Esterase (PFE, *M*_W_ = 90 kDa) resulting in a protein with a high thermal and chemical stress tolerance.^50^ PFE functions as a hydrolase in its trimeric form but loses its activity upon complex dissociation or aggregation.^51,52^ Among the different tested architectures, a PFE complex with two cross-linkers (named p4_3_Ta_2_), creating compact bicyclic structure, showed highest stability,^50^ however, it is unclear how cross-linking precisely impacts protein unfolding.

The use of IM-nMS as a collision-induced activation (CIA) or collision-induced unfolding (CIU) platform has emerged as a prevalent approach to studying protein stability and unfolding. By gradually increasing the internal energy of proteins in a controlled manner, this approach induces structural activation while preserving covalent bonds.^27,53–55^ As a result, IM-nMS techniques are well-suited for investigating protein dynamics, unfolding, and complex dissociation. TIMS offers several advantages as a platform for CIA and/or CIU analysis, as it provides a highly controlled environment for protein unfolding by the use of unique intermediate trapping and separation mechanisms that enhances resolution and sensitivity. This allows for the detection of subtle conformational changes and improved differentiation of intermediate states.^56^ The role of different TIMS potentials in activating protein structures have been studied.^57–60^ Borotto et al.^53^ applied CIU to globular proteins such as cytochrome c, myoglobin, and fc-Fusion proteins, confirming that increasing Δ6 potentials and TIMS tunnel pressure strongly drives unfolding and complex dissociation, reflected in CCS charges.^56^

In this study, we present a novel low-flow SEC-TIMS-MS platform to evaluate the stability of gas phase structures of the PFE enzyme and its chemically cross-linked variant p4_3_Ta_2_. A suite of advanced, hyphenated mass spectrometry techniques has been used to probe how chemical cross-linking influences protein structure, unfolding, dissociation, and aggregation. First, low-flow SEC was used to separate the native trimer and cross-linked trimer protein complexes from potential aggregation products and exchange to desalting buffers prior to MS analysis. Second, the TIMS-MS technique was employed to separate, analyze, and activate the native conformations of the proteins. In the latter approach, the TIMS platform also functions as a means for CIA, where the DC voltages, ramp time, and tunnel-in pressure are carefully adjusted to induce controlled unfolding and/or dissociation of the protein complex. Finally, trapped ion mobility was used to probe the native and activated structural conformation of the enzyme and its chemical cross-linked complex. Our findings reveal structural differences in protein complex stability and dissociation behavior, suggesting that chemical cross-linking plays an essential role in preserving higher-order structures and preventing aggregation. Moreover, this approach facilitates high-throughput analysis while providing insights into protein structures, rendering it a powerful addition to the structural biology toolbox.

## Experimental Section

### Sample Preparation

The PFE enzyme and the chemical cross-linked p4_3_Ta_2_ variant were prepared as previously described^50^ and stored at −80 °C in a formulated buffer (50 mM HEPES; 50 mM NaCl; pH 8). Protein solutions were diluted to 80 μM in 50 mM ammonium acetate (BioUltra; 5 M in Milli-Q H_2_O (Sigma-Aldrich), prior to analysis. Milli-Q water was obtained from the Milli-Q Direct Water Purification System (Merck Millipore).

### SEC Separation

The SEC-UV and SEC-MS experiments were performed using a UltiMate3000 UHPLC system from Thermo Fisher Scientific equipped with an UltiMate WPS-3000TFC Analytical autosampler, UltiMate NCS-3500RS Nano pump system and an UltiMate VWD-3400RS detector. The SEC column TSKgel SuperSW3000 (1.0 mm I.D. × 30 cm, 4 μm) was purchased from TOSOH Bioscience. The mobile phase consisted of 150 mM AA with a flow rate of 16 μL/min. The injection volumes were set to 3 μL and UV detection was set at 280 nm.

### Trapped Ion Mobility Mass Spectrometry Including CIU Experiments

The SEC setup was coupled online to a timsTOF Pro 2 (TIMS-Qq-TOF, Bruker) equipped with an Electrospray Ionization (ESI) source, operating in positive ion scan mode with a mass scan range of 1000–8000 *m*/*z*. Two different sets of tuning parameters, named the “standard method” and “soft method”, were used in this study (for specific parameters see Table S1). A tunnel-in pressure of in the order of 2.6 mbar was used as standard for all measurements. The instrument was calibrated on the tunnel-in pressure used during the measurements for mass and mobilities using the Agilent ESI tune mix prior to all analyses. All mobility spectra and CIU fingerprints were obtained with a ramp time of 100 ms.

To activate the protein complexes in the CIU experiments, initially the Δ6 was increased from 50 to 150 V (increments of 20 V) and 200 V, Δ1 from 0 to −284 V (max software) with 50 V steps and Δ3 from 50 to 200 V (increments of 20 V) followed by 50 V steps to 500 V. Furthermore, combinations of the various Δ-potentials have been tested, namely, combinations of two Δ-potentials, namely (i) Δ1 & Δ6 included Δ1 set to −100 or −150 V with increasing Δ6 from 70 to 200 V, (ii) Δ3 & Δ6 included Δ3 set to 110, 150, or 170 V with increasing Δ6 from 70 to 200 V, followed by (iii) varying three Δ-potentials (Δ1 & Δ3 & Δ6) where Δ1 set to −100 or −150 V, Δ3 to 110, 150, or 170 V and Δ6 at 150 or 200 V. For these experiments, the tunnel-in pressure was set between 2.6 and 1.7 mbar with a mobility range of 0.9–1.78 V·s/cm^2^ for 2.6 mbar, 0.5–1.78 V·s/cm^2^ for 2.2 and 2.0 mbar and 0.5–1.82 V·s/cm^2^ for 1.7 mbar. A full overview of the CIU experiments is presented in Table S1.

### Data Analysis

The SEC-IM-nMS data was processed using the Bruker Compass DataAnalysis (V6.1). Mobility spectra were manually exported and plotted with CIUSuite 3.^61^ The data was smoothed with the Savitzky-Golay algorithm with a window length of 5 and polynomial order of 2. To calculate the collision-cross section, mobility spectra were fitted using the multiple peak fitting tool in Fityk (V 1.3.1) and the average centroid of each fitted peak was reported.

## Results and Discussion

### SEC-UV-IM-MS under Nonstressed Conditions

We investigated the enzyme both in its wild-type (PFE) and in its cross-linked form (p4_3_Ta_2_). To enable covalent cross-linking, two solvent-exposed cysteine residues (T3C and Q174C) were introduced near the central axis of the trimer, producing the ‘p4’ variant. These cysteine sites react with the tris electrophilic reagent (Ta–I3), resulting in the formation of a compact bicyclic structure through dual-site modification, thereby effectively stabilizing the complex. Notably, both proteins predominantly exist in a homotrimeric form (Figure 1A).^62^

**Figure 1:**
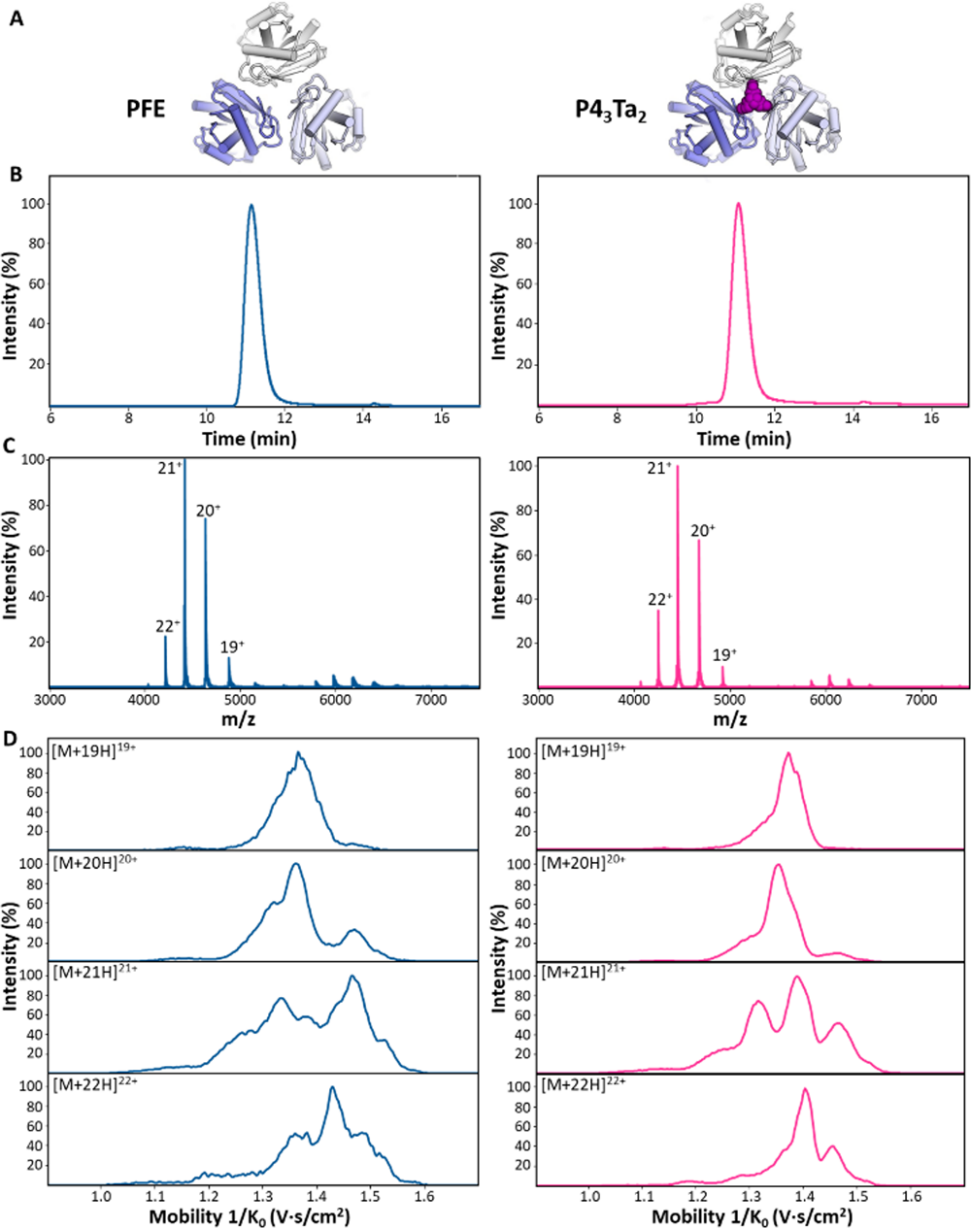
SEC-UV-TIMS-MS of PFE (blue) and p4_3_Ta_2_ (pink). (A) Structural representation of homotrimeric PFE (left) and the cross-linked PFE complex p4_3_Ta_2_ (derived from PDB ID 8PI1). (B) SEC-UV of PFE and p4_3_Ta_2_ resulted in one peak corresponding to the trimer form of the proteins. (C) SEC-MS of PFE and p4_3_Ta showing a CSD of +19 to +22 ions representing a folded structure of the proteins. (D) TIMS-MS of PFE and p4_3_Ta_2_ for the four main charge states under soft conditions to avoid activation in the gas phase.

To investigate whether CXL of protein complexes affects the protein conformation, stability, and potential unfolding in the gas phase, PFE and p4_3_Ta_2_ were analyzed using a hyphenated approach combining low-flow SEC-UV-TIMS-nMS. Since the proteins are stored in a nonvolatile buffer, native SEC was employed to desalt, separate potential aggregates, and to buffer exchange to ammonium acetate (AmAc). For that purpose, a low-flow SEC column was used providing several advantages compared to conventional SEC, namely (i) the low flow rate (16 μL/min) enhances ionization, particularly for large proteins and (ii) the reduced flow minimizes the shear force applied during separation, significantly lowering the risk of protein unfolding before MS analysis.^63^ Subsequently, both protein complexes were subjected to low-flow SEC-TIMS-MS analysis.

Figure 1B presents the SEC-UV chromatograms of PFE and p4_3_Ta_2_, showing a single peak with a retention time (RT) of 11.1 min. This RT corresponds to a protein with a molecular weight in the order of 90 kDa, based on a calibration curve of standard proteins (see Figure S1). The SEC-UV showed analogous elution behavior and peak shape for PFE and p4_3_Ta_2_ under native conditions, with similar peak areas. Hyphenating SEC with MS revealed a folded trimeric structure with a charge state distribution (CSD) centered around [M + 21H]^21+^, as shown in Figure 1C. Deconvolution yields a molecular weight of 92781.9 Da for WT-PFE (mass error = 4.8 ppm) and 93470.40 Da for p4_3_Ta_2_ (mass error = 5.4 ppm). This confirmed the presence of the three protein monomers (p4) that reacted with two linkers. A hexamer structure was also observed for both proteins at *m*/*z* = 6000 (intensity <5%).

To assess whether the protein with and without the two linkers displays similar behavior under nonstressed conditions, we adjusted the transfer DC voltages, specifically Δ1, Δ3, and Δ6, to −50, 50, and 50 V, respectively (Figure 1C) minimizing the potential activation of the enzymes by the instrument. Even under “soft conditions” the mobility behavior is more defined for the cross-linked protein complex (p4_3_Ta_2_). PFE exhibits broader and more complex peak patterns, indicating the presence of multiple conformations and enhanced conformational dynamics. For each charge state, the mobility traces reveal at least two distinct conformational families; one associated with a more compact (folded) form and another corresponding to a more extended conformation. While the more resolved structures are likely originating from solution, the peak broadness suggests that unmodified PFE has a significant structural flexibility and shows dynamic behavior in the gas phase. Cross-linked p4_3_Ta_2_ on the other hand, shows sharper, more-defined peaks. The reduced complexity and narrower distribution indicate a more rigid and conformationally restricted structure, presumably due to restricted unfolding and the ‘forced’ trimer structure in the bicyclic arrangement of p4_3_Ta_2_. To assess whether our “soft” settings preserve native-like structure, we benchmarked the experimental CCS values against EHSS (exact hard-sphere scattering) predictions from the crystal structure. The mean deviation is 4.3% for WT-PFE and 3.7% for p43Ta_2_, which lies within the typical combined uncertainty of model and calibration (≤5%). This indicates that both PFE and the cross-linked trimer are measured under conditions that retain native-like structures, with no systematic compaction (Table S2). To determine whether these extended conformations could be minimized, we further reduced Δ6, the most influential activation parameter, to 30 V, the lowest setting permitted by the software. Despite this adjustment, similar conformations were observed (Figure S2).

### Probing Protein Stability and Structure via Collision-induced Activation

Collision-induced activation experiments were performed using TIMS, see Figure 2A. For these experiments, ions are trapped inside the TIMS tunnel by gradually increasing the electric field in combination with a flowing gas (Figure 2B). The ions are then separated based on their mobility by decreasing the electric field in the second part of the tunnel (named Analyzer 2 in Figure 2A). Extended ions elute first as they experience greater force from the gas, while compact ions elute later. The mobilities are plotted as reduced mobility 1/*k*_0_, where a lower value corresponds to a more compact structure and a higher value to a more extended structure. TIMS allows to tune resolution by adjusting the duration of the mobility scan. Key parameters that influence ion activation in the gas phase include a series of Δ potentials, tunnel gas pressure, and accumulation time, which can be precisely controlled. Figure 2A provides a schematic representation of the TIMS setup and the locations of each of the Δ potentials within the instrument.

**Figure 2:**
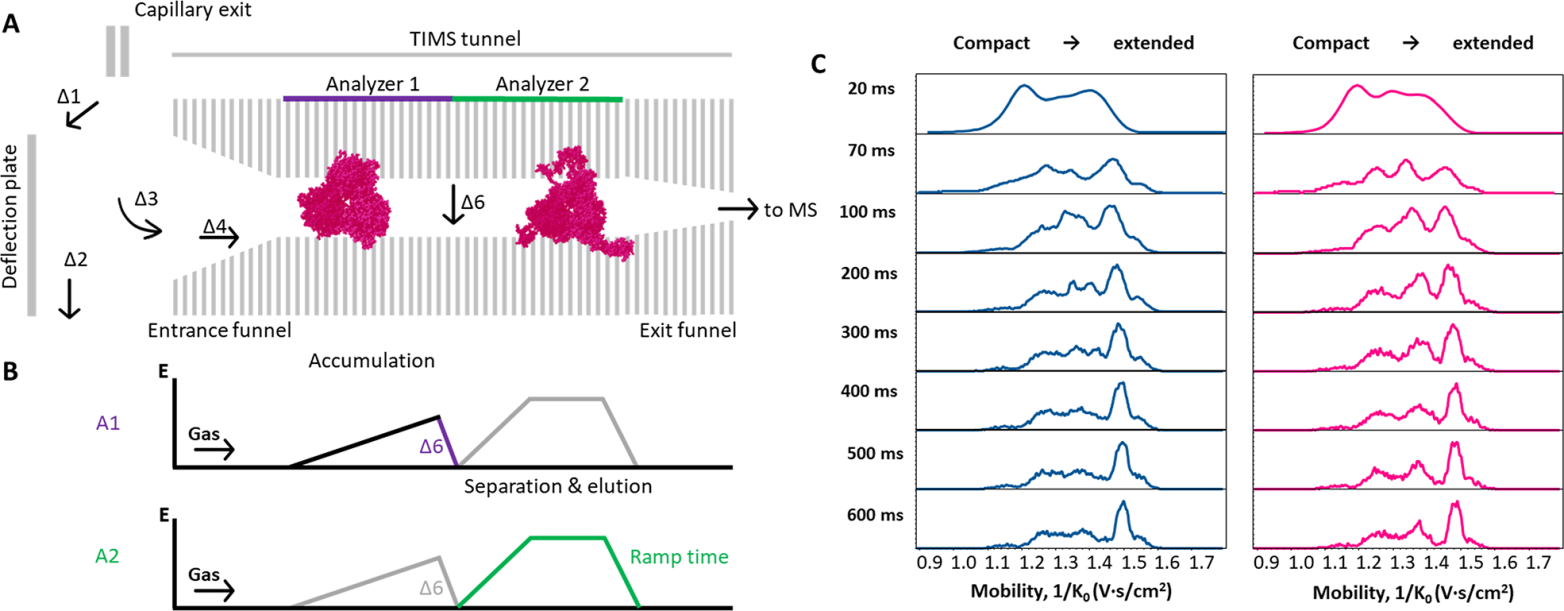
(A) Schematic overview of TIMS instrument with the location of all Δ-potentials which can affect CIU. (B) Electric field applied during TIMS accumulation (A1) and separation (A2) with the location of Δ6 DC voltage indicated. (C) Mobility spectra of the [M + 21H]^21+^ charge ion of PFE (blue) and p4_3_Ta_2_ (pink) measured at ramp time of 20 to 600 ms.

To optimize the resolution of the conformational states and unfolding transitions, we systematically increased the TIMS ramp time, which is a key parameter for improving ion mobility resolution. Figure 2C shows the mobility profiles for the most abundant charge state, [M + 21H]^21+^ across varying ramp times (*t* = 20 to 600 ms) for PFE (blue) and p4_3_Ta_2_ (pink) using the standard method outlined in Table S1. At the shortest ramp time of 20 ms, conformational features remain poorly resolved due to the limited separation time and the mobility distribution displays a slight shift toward more compact features. This apparent compaction does not reflect a truly more folded structure but results from nonsteady-state elution: the field is reduced too quickly for large protein ions to reach equilibrium between drag force and electric field. This leads to premature elution at lower mobility (1/*K*_0_), giving the appearance of a more compact feature, although it is an artifact of non-steady-state elution rather than a real conformational change. As the ramp time increases, particularly between 70 and 100 ms, distinct conformers become clearly resolved for both protein species. A ramp time of 100 ms provides slightly improved separation while minimizing potential artifacts such as protein refolding, undesired gas-phase interactions, or ion decay during extended scan durations. Therefore, a ramp time of 100 ms is selected for subsequent experiments.

### Monitoring Unfolding Pathways of PFE and p43Ta2 via CIA

#### Effect of the Δ6 Potential

Δ6 is the primary parameter used to study the unfolding of proteins during collision-induced activation.^55^ To explore its impact on our large protein complexes, Δ6 was systematically increased from 50 to 130 V in increments of 20 V, followed by smaller increments of 10 V up to 200 V (max. value), while maintaining a standard tunnel-in pressure of 2.6 mbar (for values of other instrument parameters see Table S1). Figure 3A,B present the results as mobility profiles and Figure 3C,D show the heat map representations where the ^TIMS^CCS_N2_ values are plotted against increasing Δ6 voltage. We focused on the unfolding behavior of the [M + 21H]^21+^ ion of PFE (Figure 3A,C) and of p4_3_Ta_2_ (Figure 3B,D). Additional charge states and their representative CIU fingerprints are provided in Figure S3. The mobility spectra for PFE (in blue) reveal the presence of a broad distribution of conformations at low Δ6 voltages (Δ6 < 130 V), multiple structures are detected between 1/*K*_0_ = 1.1–1.5 V·s/cm^2^ with the main peak centered at 1/*K*_0_ = 1.4 V·s/cm^2^. As the activation voltage increases beyond 160 V, these conformations collapse into a single dominant, extended conformation peaking at 1/*K*_0_ = 1.48 V·s/cm^2^. The CIU data, presented in Figure 3C, is consistent with these conformational changes. At Δ6 values up to 130 V, multiple compact structures are detected with ^TIMS^CCS_N2_ values ranging from 5300–6300 Å^2^, suggesting a variety of stable, folded conformers. Between 140 and 170 V, a clear transition occurs, with a shift toward more extended conformations. At 200 V, PFE adopts a narrow ^TIMS^CCS_N2_ distribution centered around ∼6500 Å^2^ consistent with a fully unfolded structure.

**Figure 3:**
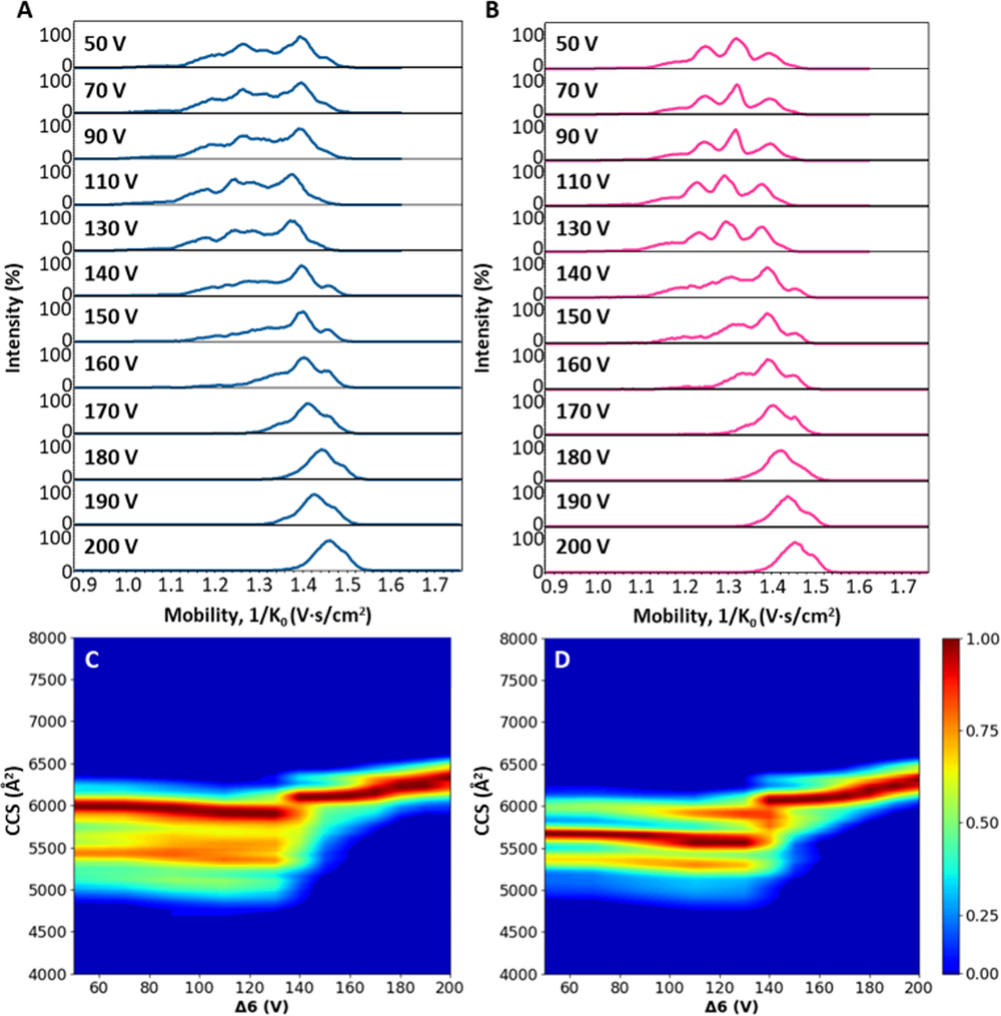
(A) Extracted mobility spectra PFE in blue of [M + 21H]^21+^ with increasing Δ6 from 50 to 200 V. (B) Extracted mobility spectra in pink of p4_3_Ta_2_ [M + 21H]^21+^ with increasing Δ6 from 50 to 200 V. (C) CIU fingerprint PFE [M + 21H]^21+^ with CCS values when increasing Δ6 from 50 to 200 V. (D) CIU fingerprint p4_3_Ta_2_ [M + 21H]^21+^ with CCS values when increasing Δ6 from 50 to 200 V.

The mobility spectra of the p4_3_Ta_2_ complex, in pink in Figure 3B, show higher-resolved conformations at Δ6 = 50–130 V. The mobility spectra reveal four main peaks with 1/*K*_0_ = 1.20, 1.27, 1.34, and 1.42 V·s/cm^2^ from the most compact to the most extended conformation. Similar to PFE, with increasing Δ6 voltages, a shift toward reduced conformational diversity and extended structures is observed where the most intense peak corresponds to 1/*K*_0_ = 1.48 V·s/cm^2^. This is reflected in the CIU plots, where up to 140 V, the structural profile remains similar to PFE but with lower CCS values ^TIMS^CCS_N2_ = 5700 Å^2^, despite the increase in the molecular weight.

Both PFE and p4_3_Ta_2_ begin to unfold around Δ6 = 140 V and reach extended conformations at 200 V. However, consistently smaller CCS values are observed for the p4_3_Ta_2_, indicating more compact intermediate states. While the activation threshold (Δ6 = 140 V) is similar across charge states (Figures S3.1–S3.3), the two proteins follow distinct unfolding pathways. This difference likely originates from the structural stabilization in p4_3_Ta_2_, where the two cross-linkers anchor the trimer at both the N- and C-terminal ends, helping it retains compact conformations at intermediate voltages. Despite these differences, both proteins ultimately reach similar extended states. This is most likely due to the large size of these protein complexes limits the effect of Δ6 at 2.6 mbar, resulting in only partial unfolding. This suggests that for large protein complexes, combined activation strategies, such as preactivation using for example Δ3/ Δ1 potential and/or lower tunnel-in gas pressures will be necessary to probe more significant structural variations.

#### Influence of Tunnel-In Pressure

In TIMS, the ions are subjected to a combination of electric fields and the pressure of the gas (N_2_) that guides the ions into the TIMS cartridge. As the pressure is lowered, the gas-phase environment becomes less dense, which affects how ions interact with the background gas. When ions collide with fewer gas molecules, i.e. lower tunnel-in pressure, they retain more of their kinetic energy and can undergo greater unfolding.^64^ Here, we examined how decreasing the tunnel-in pressure influences protein conformation while systematically increasing the Δ6 voltage in 20 V increments. Tunnel-in pressures of 2.2, 2.0, and 1.7 mbar were explored for PFE and p4_3_Ta_2_ alongside the standard tunnel-in pressure of 2.6 bar, which was used in Figure 3. We then obtained CIU fingerprints at different pressures, thereby focusing on the analysis of charge state [M + 21H]^21+^ (Figure 4). The CIU fingerprints of the additional charge states can be found in Figures S4.1–S4.4.

**Figure 4:**
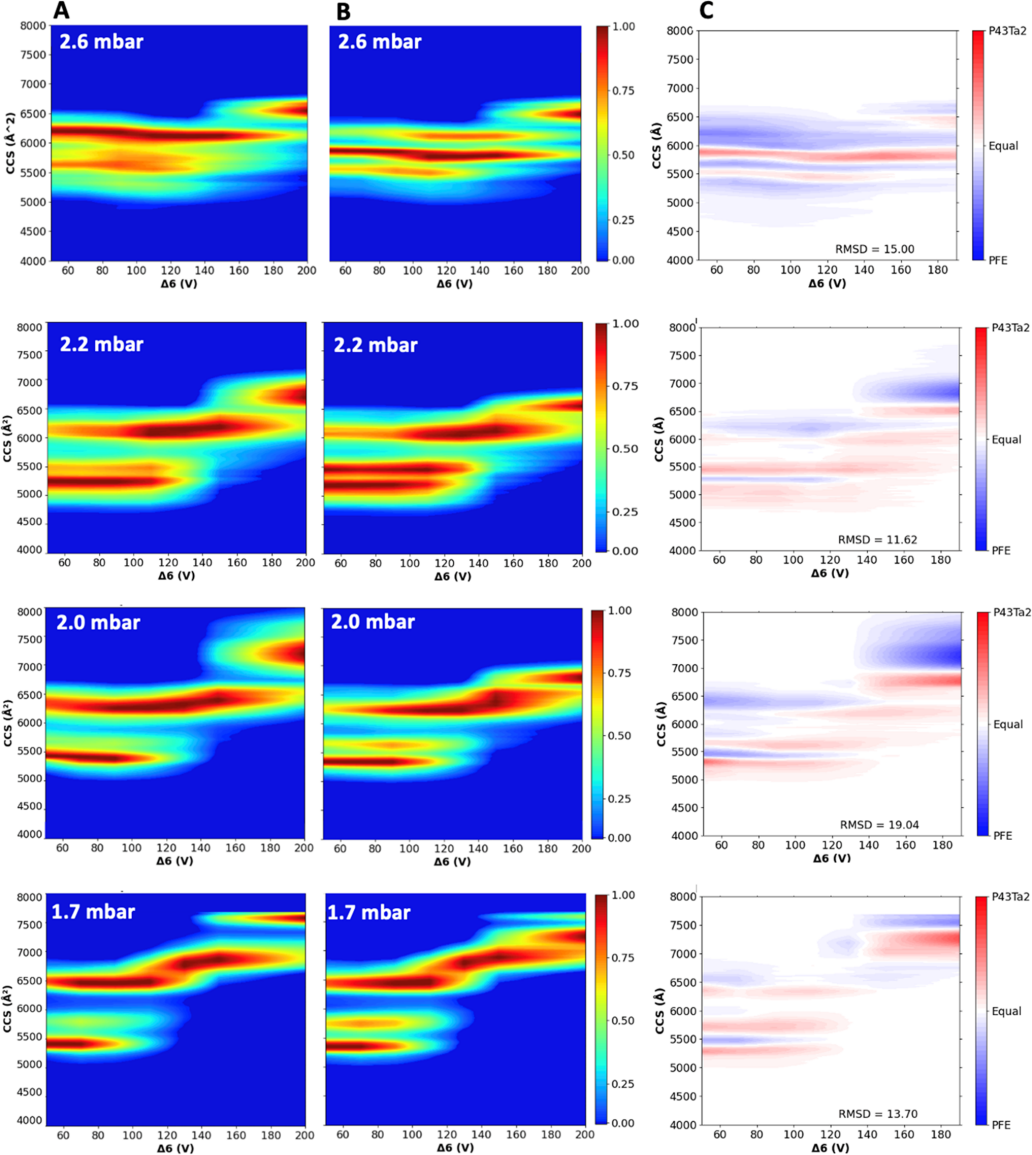
(A) CIU fingerprint for PFE [M + 21H]^21+^ at the tunnel-in pressure of 2.6, 2.2, 2, and 1.7 mbar with CCS values when increasing Δ6 from 50 to 200 V. (B) CIU fingerprint for p4_3_Ta_2_ [M + 21H]^21+^ at the tunnel-in pressure of 2.6, 2.2, 2, and 1.7 mbar with CCS values when increasing Δ6 from 50 to 200 V. (C) CIU comparison plot analysis depicting PFE and p4_3_Ta_2_ at different tunnel-in pressure with Δ6 on *x*-axis, CCS value on *y*-axis, and color scheme representing the differential intensities of PFE (Blue) and p4_3_Ta_2_ (red).

The CIU profile of PFE at 2.2 mbar reveals differences compared to the standard pressure of 2.6 mbar. The primary unfolding transition occurs still around a Δ6 of 140 V, but the structural resolution is significantly improved at the lower pressure of 2.2 mbar, illustrated by more-defined mobility peaks, and shifted intensity to more compact structures (with ^TIMS^CCS_N2_ values of 5200 and 5500 Å^2^). At 2.6 mbar, the dominant conformation of PFE is already extended (^TIMS^ CCS_N2_ ∼ 6200 Å^2^) and begins to further open (^TIMS^CCS_N2_ = 6800 Å^2^). In contrast, at 2.2 mbar, the compact conformer is mostly present and shifted to a more compact structure around 5300 Å^2^, and the unfolding pathway becomes more finely resolved. Additionally, the extended conformers become more pronounced at 2.6 bar, with CCS extending further, from ∼6700 to ∼7000 Å^2^, indicating greater conformational expansion under these conditions.

p4_3_Ta_2_ also shows increased compactness at 2.2 mbar, exhibiting two distinct and well-resolved conformers at ^TIMS^CCS_N2_ = 5200 and 5500 Å^2^. Although, the extended structures remain in the same range ^TIMS^CCS_N2_ = 6800 Å^2^, the overall unfolding pathways shows a distinct pathway which is not observed in the 2.6 mbar plot. This indicates that the cross-linker in p4_3_Ta_2_ enhances its structural rigidity, thereby presumably restricting the number of possible unfolding pathways and intermediates. The right panel shows the CIU comparison plots, where blue indicates PFE and red p4_3_Ta_2_ (Figure 4C). The CIU comparison plot at 2.2 mbar between the two proteins clearly highlights how PFE adopts to a wider array of extended conformers (RMSD = 11.62), reaching up to 7800 Å^2^, compared to the conformationally constrained *P*4_3_Ta_2_.

At a reduced tunnel-in pressure of 2.0 mbar, PFE exhibits less well-defined compact structures compared to those observed at 2.2 mbar. Although a compact population remains centered around 5400 Å^2^, the most dominant population is more extended with ^TIMS^CCS_N2_ = 6300 Å^2^. Moreover, the transition to extended conformations becomes less defined, as the intermediate states appear less resolved. The extended forms of PFE, observed at Δ6 > 140 V, span over a broad range of CCS values from approximately 7000 to 8000 Å^2^. This indicates a more heterogeneous and less structured unfolding profile compared to those seen at higher pressures (>2.2 mbar). In contrast, p4_3_Ta_2_ shows consistent structural compactness under decreasing pressures, i.e., retaining conformers within a similar CCS range (ca. 5400 Å^2^), although unfolding proceeds in a more stepwise and orderly fashion. Even in its most extended form, p4_3_Ta_2_ remains similar to those observed at higher pressures ^TIMS^CCS_N2_ = 6800 Å^2^ rather than the broad, unfolded distribution observed for PFE. The CIU comparison plot show a RMSD of 19.04, indicating that, at this tunnel-in pressure, the proteins exhibit significant differences in their unfolding profiles and the pathways they follow. This is clearly visible at CCS values between 7000 and 8000 Å^2^, where the PFE complex shows extensive unfolding while p4_3_Ta_2_ retains structures below 7000 Å^2^.

At the lowest pressure of 1.7 mbar, for PFE, a new population of extended conformers emerges around 7000 Å^2^ upon activation with a Δ6 of 120 V. In addition, a final extended conformer appears around 7800 Å^2^ that is well-defined and narrowly distributed, representing the most distinct and highly extended form observed across all tunnel-in pressures. Similarly, p4_3_Ta_2_ exhibits a new population of extended conformers activated at 120 V. However, the majority of its extended conformers remain centered around 7500 Å^2^, with the fully extended conformation at 7800 Å^2^ appearing only at very low intensity. Despite some similarity, CIU comparison (RMSD = 13.76) reveals clear differences between the unmodified and the cross-linked protein complex, particularly in the extent and distribution of extended conformers. Across WT-PFE charge states (19+ to 22+) we observe clear Coulombic destabilization: at 19+ a dominant compact population (∼4900–5300 Å^2^) persists, but as charge of the protein complex and Δ6 voltage increase, the compact band progressively destabilizes. and multiple intermediate states emerge. This can most prominently be observed for the 20+ and 21+ charge state, indicating a less cooperative, more heterogeneous unfolding pathway). For the 22+ charge state, the shift toward extended conformations appears at a lower activation voltages and have higher CCS values. In contrast, the cross-linked trimer (p43Ta_2_) resists this charge-induced unfolding: across 19+ to 22+ the compact conformer (∼5100–5600 Å^2^) remains dominant, conformers with higher CCS values are weak and only appear at high Δ6 voltages. This results in CCS distributions that are narrower indicating that fewer intermediate conformations are present. Even at 22+, the complex stays largely compact with no dramatic transition to extended states. Together, our data show that while WT-PFE undergoes charge-driven (Coulombic) destabilization, but cross-linking suppresses this process by raising the activation threshold and attenuating unfolding transitions. Overall, at 2.2 mbar, PFE undergoes extensive unfolding, reaching up to 8000 Å^2^, while p4_3_Ta_2_ remains relatively compact. This demonstrates that, under these tunnel-in conditions, the Δ6 activation achieves its maximum unfolding effect, distinctly revealing the differences in structural flexibility between the two protein complexes.

#### Effect of Δ1 Potential

Δ1 is the voltage applied between the capillary exit and the deflection plate (Figure 2A) and plays a key role in promoting desolvation. This is especially important for large proteins such as PFE and p4_3_Ta_2_, where optimized Δ1 settings (typically around −50 to −100 V) contribute to improved spectral clarity. Since the insource CID (isCID) acts as a bias voltage, creating an offset for all optics, the ions experience a voltage drop at the exit of the TIMS tunnel (entrance funnel 2). In other words, declustering or adduct removal aimed at enhancing the MS signal, occurs subsequent to ion mobility separation. For large proteins, this means that much of the signal cleanup, such as adduct removal, occurs too late to improve the quality of the mobility data. However, increasing Δ1 provides energy before the ions enter the TIMS tunnel. This helps to reduce adducts and improve desolvation before mobility separation. In addition, Δ1 enables charge-dependent activation,^59^ allowing the investigation of protein unfolding for a specific charge state.

To characterize structural changes in PFE and p4_3_Ta_2_, the Δ1 voltage was incrementally increased from 0 to −150 V (50 V steps, see Table S1 for the other parameters). This range represents the upper limit for Δ1, as signal intensity drops off sharply beyond −150 V, and no detectable signal is observed at −200 V. Figure 5 presents the overlay of mobilities for PFE and p4_3_Ta_2_ across the 22+ to 19+ charge states. For [M + 19H]^19+^, which is the most compact charge state for both proteins, increasing Δ1 up to −150 V consistently enhanced desolvation and improved the resolution of compact structures without indications of unfolding for both proteins. Also for [M + 20H]^20+^, both protein complexes behave similarly, where the Δ1 voltage initially increases compact structural resolution between 0 and −50 V, but from −100 to −150 V, the proteins start to unfold, and extended structures appear. At −150 V, the extended structures dominate the mobility spectra, with PFE exhibiting a more unfolded structure than p4_3_Ta_2_.

**Figure 5:**
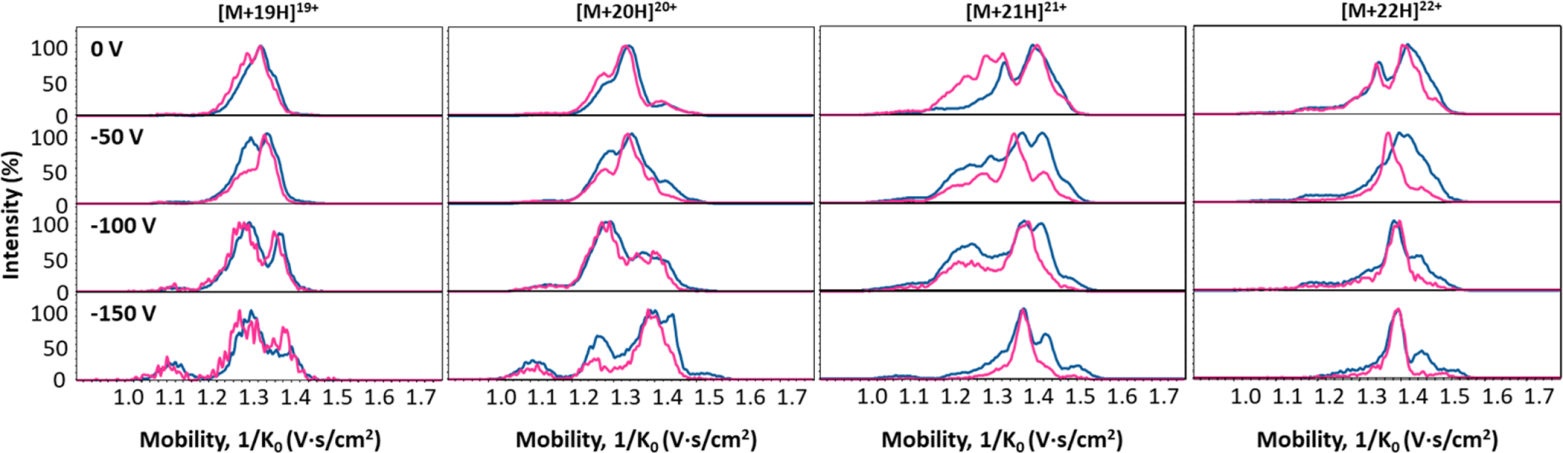
Overlay of extracted mobility spectra PFE (blue) and p4_3_Ta_2_ (pink) with increasing Δ1 from 0 to −150 V for the four charge states. For charge starts of [M + 19H]^19+^ and [M + 20H]^20+^, both proteins behave similarly with the increase in Δ1 enhancing the compact structure resolution. For [M + 21H]^21+^ and [M + 22H]^22+^, PFE shows unfolding behavior starting at −50 V and an increase in the extended structures while p4_3_Ta_2_ holds the more compact structures.

For [M + 21H]^21+^ PFE and p4_3_Ta_2_ display distinct behaviors. PFE begins to unfold at relatively low Δ1 values, as observed by the mobility peaks above 1/*K*_0_ = 1.4 V·s/cm^2^, starting already from −50 V. In contrast, p4_3_Ta_2_ maintains a more compact conformation as Δ1 voltage increases. Interestingly, at low Δ1 values (e.g., 0 V), p4_3_Ta_2_ exhibits greater conformational diversity of the compact structures than PFE in the 21+ charge state. These extra conformations may be due to the structural rigidity introduced by the cross-linker, which slows down the interconversion between different conformations, thereby preserving a broader population of distinct compact conformers that are not averaged out by conformational interconversion. For [M + 22H]^22+^, PFE begins to unfold between 0 and −50 V and continues to unfold until −150 V. P4_3_Ta_2_, retains a more compact conformation throughout the Δ1 voltage range, in line what was observed for the 19+ and 20+ charge states. The CIU fingerprints of PFE and p4_3_Ta_2_ are summarized in Figure S5. Besides facilitating desolvation, the effect of Δ1 is charge-dependent, where for more extended conformations even low levels of Δ1 can promote unfolding. We also observe that increasing Δ1 leads to more unfolding and structural extension in PFE, while p4_3_Ta_2_ remains stable and is less susceptible to unfolding under the same conditions.

#### Effect of Δ3 Potential

The Δ3 potential represents the voltage applied to steer the ions into the TIMS tunnel, see Figure 2A. Since high Δ3 values can also influence protein stability and conformation,^59^ Δ3 was incrementally increased from 50 to 450 V, and its effects on protein unfolding were analyzed. Both PFE and *P*4_3_Ta_2_ exhibited similar mobility profiles as Δ3 increased (Figure S6). For charge states [M + 19H]^19+^ and [M + 20H]^20+^, activation only occurred at high Δ3 values (above 350 V). For the higher charge states [M + 21H]^21+^ and [M + 22H]^22+^, both proteins showed two distinct activation points, namely at 170 and 350 V. Although the intermediate structures are differed between PFE and p4_3_Ta_2_, the final extended structures have similar CCS values (6520.5 and 6679.8 respectively). Overall, the effect of Δ3 on unfolding of both protein complexes was limited, and only at extremely high voltages significant conformational changes were induced. This behavior is likely due to the large size of the protein complexes, as Δ3 has a more pronounced effect on smaller proteins.^59^ It can be argued that Δ3 alone is not sufficient for preactivation and that very high voltages are needed to activate these structures. Additionally, the combination of Δ3 with other Δ*v*oltages (Δ1 or Δ6) was evaluated related to protein stability. The combination of Δ1 with Δ3 yielded no additional unfolding effects beyond those caused by Δ1 alone. Similarly, when Δ3 was combined with Δ6, the unfolding behavior was largely dominated by Δ6. An overview of the mobilograms under these parameter combinations is provided in Figures S7.1 and S7.2.

### Investigation of Gas-Phase Dissociation Patterns in PFE and p4_3_Ta_2_

In addition to examining unfolding behavior, we analyzed the dissociation patterns of both protein complexes (PFE and *P*4_3_Ta_2_) to evaluate whether increasing Δ-voltage could induce dissociation into their monomeric subunits. Specifically, we ramped up the Δ1 and Δ6. However, under these conditions, neither complex showed dissociation into monomers (Figures S8 and S9). Interestingly, a different behavior emerged when the Δ3 voltage was increased. To explore this further, we collected mobility data at a Δ3 setting of 400 V as presented in the heat map in Figure 6A, where the *m*/*z*-values are plotted against inverse ion mobility (1/*K*_0_). In the heat map of PFE (Figure 6A, top), we observed a distinct cluster of ions in the *m*/*z* range of 1000–2000 (highlighted by the white box), which corresponds to the monomeric subunits of the complex. This indicates that at high Δ3 voltage, the PFE complex undergoes gas-phase dissociation, releasing monomer units that can be detected as separate species based on their *m*/*z* and mobility profiles. The distribution of monomer signals across a wide range of inverse mobilities suggests that these subunits are structurally heterogeneous, likely resulting from unfolding prior to dissociation. In stark contrast, the p4_3_Ta_2_ complex (Figure 6A, bottom) does not display any signal in this monomeric *m*/*z* range, and its ion mobility profile remains relatively compact, suggesting that the complex remains intact and resists gas-phase fragmentation even under high-energy activation. This divergence in dissociation behavior between PFE and p4_3_Ta_2_ highlights the stabilizing effect by covalent cross-linking in p4_3_Ta_2_, which prevents the ejection of individual monomeric units.

**Figure 6:**
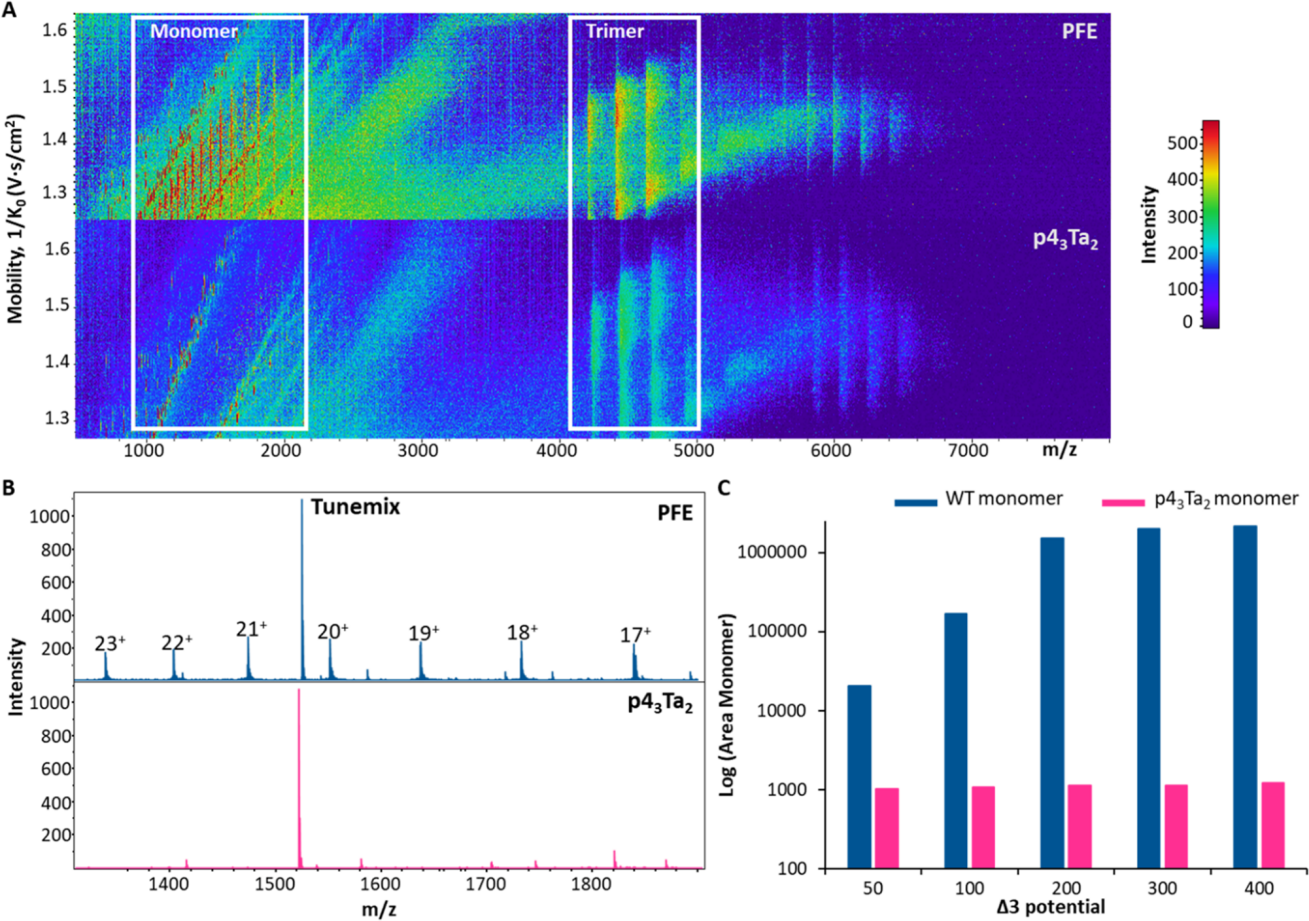
(A) Heat map of PFE and p4_3_Ta_2_ where *m*/*z* is plotted against 1/*K*_0_ for Δ3 = 400. (B) Mass spectra of PFE and p4_3_Ta_2_ between *m*/*z* 1000–2000 corresponding to the monomer units. (C) Peak area (in log) of the monomer unit for PFE and p4_3_Ta_2_ when Δ3 is increased from 50 to 400 V. *The peak labeled in graph B as Agilent tune mix corresponds to *m*/*z* = 1521.9.

To further verify this dissociation profile, we extracted and compared the mass spectra from both samples under these high-voltage conditions (Figure 6B). In the mass spectrum for PFE (top, blue), we observe a series of well-resolved peaks corresponding to the monomeric subunits, with charge states ranging from 23+ to 17+. On the other hand, the mass spectrum for p4_3_Ta_2_ (bottom, pink) shows almost no signal within the monomeric *m*/*z* window. The absence of monomer peaks strongly suggests that the cross-linked p4_3_Ta_2_ complex maintains its native trimeric form and resists dissociation even under collisional conditions that disrupt the unmodified PFE complex.

To investigate the extent of this stabilization across a range of activation conditions, we quantified the area under the monomer peaks in both complexes as a function of increasing Δ3 potential, from 50 to 400 V (Figure 6C). For unmodified PFE, we observed considerable monomer formation under all conditions, even at the lowest Δ3 value tested (50 V). In addition, the monomer signal increased up to 200 V where it reaches a plateau, which consistent with progressive destabilization and dissociation of the complex under increased stress. In contrast, the p4_3_Ta_2_ shows consistently low monomer signal across all Δ3 settings, with no significant increase in monomer even at 400 V. This resistance to dissociation confirms that cross-linking enhances the mechanical and structural robustness of the complex in the gas phase. These data demonstrate that PFE dissociates into monomeric subunits upon collisional activation, thereby losing its higher-order structural integrity. In contrast, the covalent trimer p4_3_Ta_2_ remains intact, highlighting the protective effect of chemical cross-linking against voltage-induced fragmentation. This further confirms the ability of INCYPRO cross-linking to preserve complex integrity under harsh MS conditions which is often needed for large protein transmission without concern for altering their native structure.

## Conclusions

We investigated the impact of chemical cross-linking on the structural stability of protein complexes in the gas phase using TIMS-MS comparing the unmodified wild-type enzyme PFE with its chemically cross-linked variant p4_3_Ta_2_. Both proteins were subjected to systematic collision-induced activation by varying tunnel-in pressures and the pre- and postaccumulation activation Δ*v*oltages. Our results reveal that the unmodified PFE complex undergoes extensive structural changes under increasing activation voltages, exhibiting unfolding at elevated Δ6 and tunnel-in pressures and dissociation into monomeric subunits at elevated Δ3 voltages. In contrast, p4_3_Ta_2_ demonstrated enhanced gas-phase stability. It retained a more compact and structured conformation throughout activation and did not produce dissociation fragments, even under the highest applied voltages. These results demonstrate that chemical cross-linking significantly enhances the structural resilience of protein complexes in the gas phase. This stabilization effect reduces gas-phase-related artifacts such as unfolding or dissociation, which can otherwise mask conformations in native MS and IMS analyses. Notably, the here reported high stability of p4_3_Ta_2_ in the gas-phase is in line with its earlier reported resistance toward thermal and chemical stress in solution resulting in considerably increased shelf life and activity under denaturing conditions.^50^ The ability of protein cross-linking to preserve higher-order structure appears to be a general effect supporting its utility as a tool in structural biology for studying dynamic protein assemblies. More broadly, the integration of cross-linking with advanced platforms such as low-flow SEC-TIMS-MS can enable high-throughput characterization of proteins and complexes that are typically challenging to study.

## Supplementary Material



## Data Availability

The data underlying this study are openly available in DataCite Commons at 10.48338/VU01-SRXNAG

## Notes

Information TIMS parameters used for CIU experiments; comparison of theoretical and experimental CCS values; SEC–UV chromatograms of protein standards; mobility profiles of PFE and p43Ta_2_ under soft conditions; heatmaps and mobility profiles of PFE and p43Ta_2_ at increasing Δ6 potentials; CIU fingerprints of PFE and p43Ta_2_ at different tunnel-in pressures; CIU fingerprints of PFE and p43Ta_2_ at increasing Δ1 and Δ3 potentials; extracted mobility spectra of PFE and p43Ta_2_ at Δ3 and combined Δ3- Δ6 voltages; and MS spectra of PFE and p43Ta_2_ at variable Δ1 and at maximum Δ6 activation (PDF)
