# Supplementary material for: Stabilizing Proteins by Chemical Cross-Linking: Insights into Conformation, Unfolding, and Aggregation Using Native Ion Mobility Mass Spectrometry
Source: Anal Chem. 2025 Nov 17;97(47):26302–12. doi: 10.1021/acs.analchem.5c06844 (PMC12676522; doi:10.1021/acs.analchem.5c06844)
Supplement: Supplementary file 1 [file ac5c06844_si_001.pdf]

Supplementary information

Stabilizing proteins by chemical cross-linking: insights into conformation, unfolding, and aggregation using native ion mobility mass spectrometry

Raya Sadighi,[a,b] Rosalin M. A. van Paasen,[a] George H. Hutchins,[c,d] Ivar D. Jansen,[d] Saskia Neubacher,[c] Tom N. Grossmann,*[d,e] and Anouk M. Rijs *[a,b]

a. Division of Bioanalytical Chemistry (MS-Laserlab), Department of Chemistry and Pharmaceutical Sciences, Amsterdam Institute of Molecular and Life Sciences, Vrije Universiteit Amsterdam, De Boelelaan 1085, 1081 HV Amsterdam, The Netherlands

b. Centre for Analytical Sciences Amsterdam (CASA), The Netherlands

c. Incircular B.V., De Boelelaan 1085, 1081 HZ Amsterdam, the Netherlands

d. Department of Chemistry and Pharmaceutical Sciences, Amsterdam Institute of Molecular and Life Sciences, Vrije Universiteit Amsterdam, De Boelelaan 1085, 1081 HZ Amsterdam, The Netherlands

e. Institute of Organic and Biomolecular Chemistry, Faculty of Chemistry, Georg-August-Universität Göttingen, Tammanstr. 2, 37077 Göttingen, Germany

Corresponding Authors: tom.grossmann@uni-goettingen.de - a.m.rijs@vu.nl

Table of Contents

Table S1	Page S2
Table S2	Page S2
Figure S1	Page S3
Figure S2	Page S3
Figure S3	Page S4
Figure S4	Page S7
Figure S5	Page S9
Figure S6	Page S9
Figure S7	Page S10
Figure S8	Page S11
Figure S9	Page S11

Table S1. Overview of all TIMS parameters, including changes in CIU experiments; -: settings were not changed, and soft values were used.

Type of experiment	Δ1 (V)	Δ2 (V)	Δ3 (V)	Δ4 (V)	Δ5 (V)	Δ6 (V)	Accumulation time (ms)	Ramp time (ms)
Soft method	-50	-150	50	100	0	50	50	100
Δ6	-	-	-	-	-	70, 90, 110, 130, 140, 150, 160, 170, 180, 190, 200	-	-
Δ1	0, -100, -150, -200, -250, -284	-	-	-	-	-	-	-
Δ3	-	-	60, 70, 90, 110, 130, 150, 170, 190, 200, 250, 300, 350, 400, 450, 500	-	-	-	-	-
Ramp time	-	-	-	-	-	-	-	20, 200, 300, 400, 500, 600
Δ1 & Δ6	-100, -150	-	-	-	-	70, 100, 150, 200	-	-
Δ3 & Δ6	-	-	110, 150, 170	-	-	70, 100, 150, 200	-	-
Δ1 & Δ3 & Δ6	-100, -150	-	110, 150, 170	-	-	150, 200	-	-

Table S2. Overview of theoretical CCS values based on PA and EHSS-like models versus the experimental CCS calculated for +19.

Name	Mean CCS (PA)	CCS (EHSS-like) (≈+8%)	Experimental CCS +19
PFE	4,540 Å²	4,903 Å²	5,117 Å²
Pa4Ta3	4,549 Å²	4,913 Å²	5099 Å²

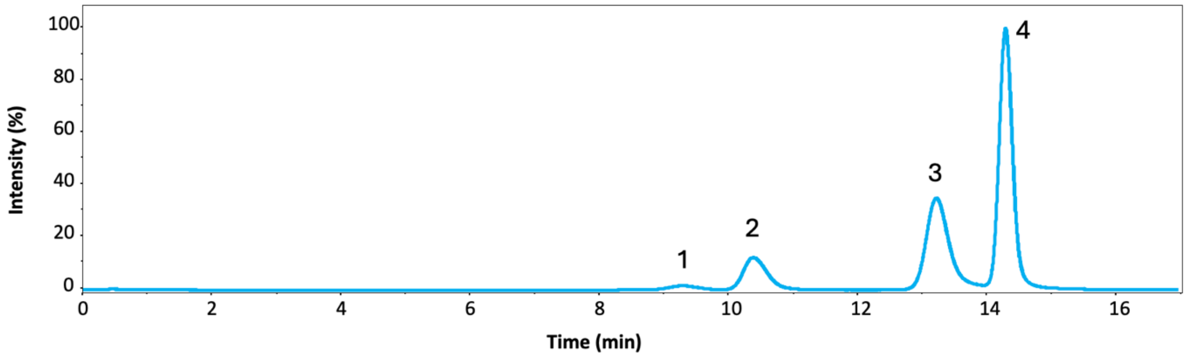

Figure S1. SEC-UV of the protein test mix containing BSA dimer (1), BSA monomer (2), cytochrome C (3), and uracil (4) using an eluent of 150 mM ammonium acetate.

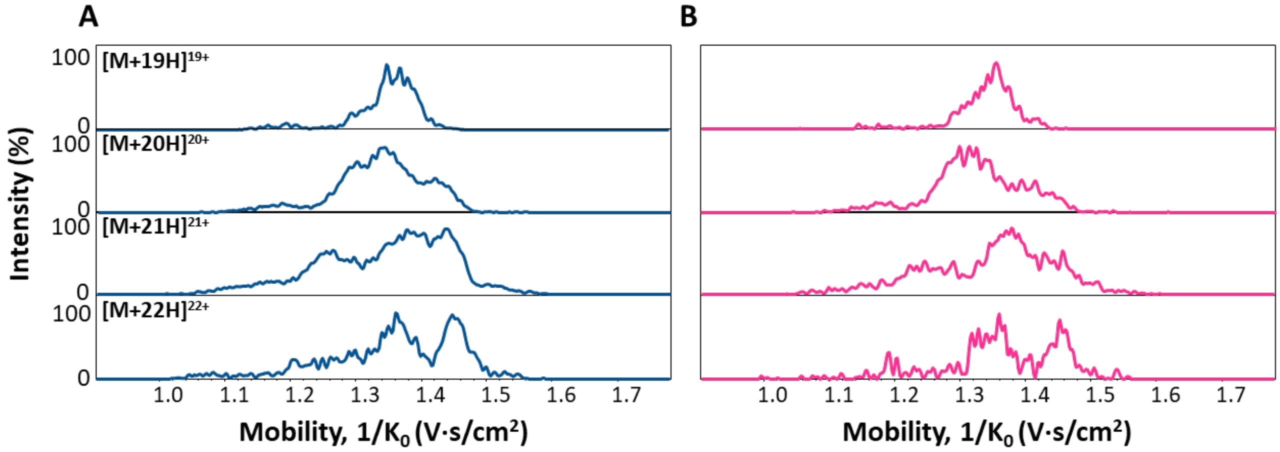

Figure S2. **(A)** Extracted mobility spectra of PFE with Δ6 at 30 V for $[M+19H]^{19+}$, $[M+20H]^{20+}$, $[M+21H]^{21+}$ and $[M+22H]^{22+}$. **(B)** Extracted mobility spectra $p4_3Ta_2$ on Δ6 at 30 V for $[M+19H]^{19+}$, $[M+20H]^{20+}$, $[M+21H]^{21+}$ and $[M+22H]^{22+}$.

Figure S3.1 to S3.3: Extracted mobility spectra and CIU fingerprints of PFE and $p4_3Ta_2$ for $[M+19H]^{19+}$, $[M+20H]^{20+}$, $[M+22H]^{22+}$ when increasing Δ6 from 50 to 200 V.

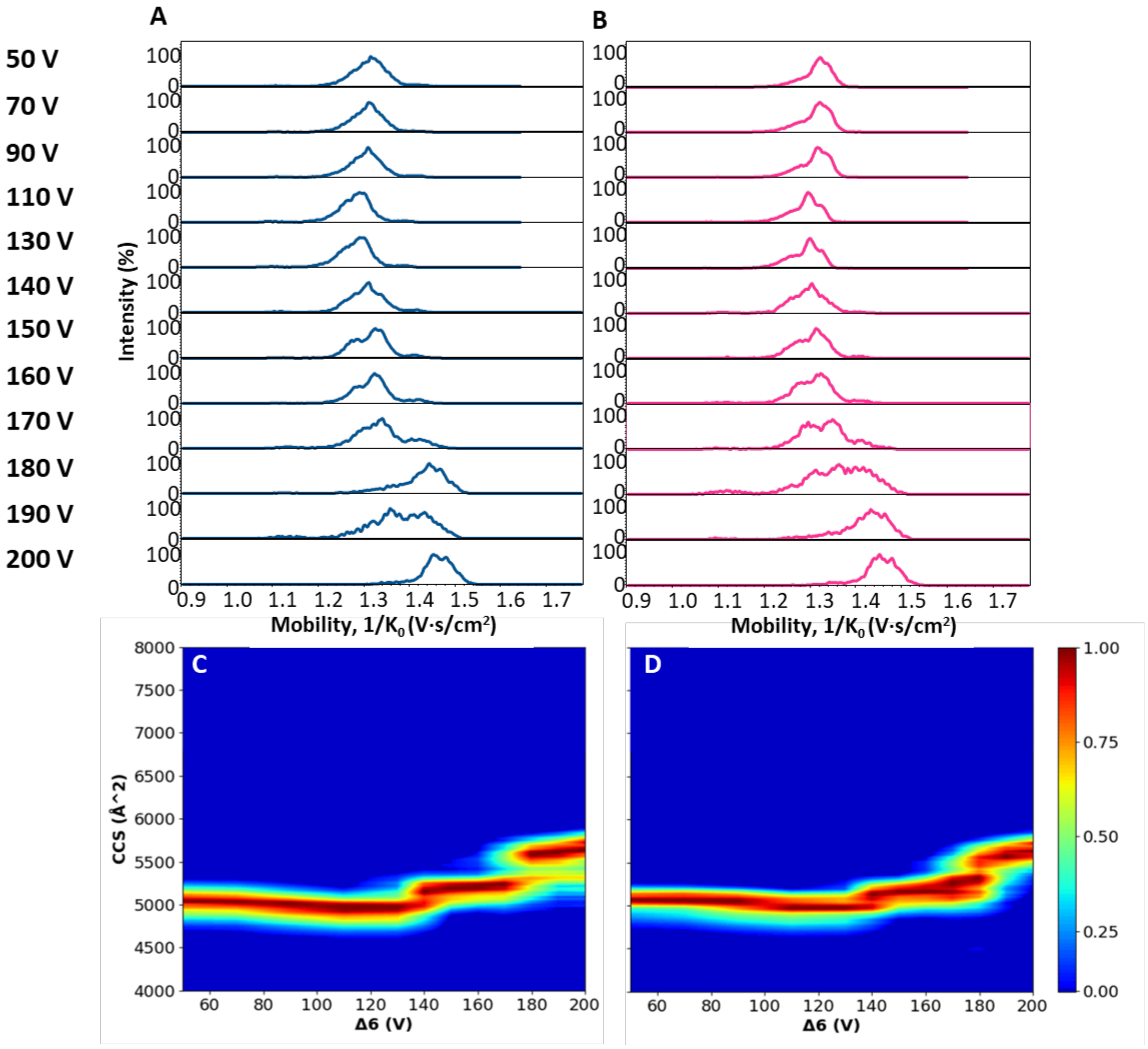

Figure S3.1 (A) Extracted mobility spectra of PFE (blue) **(B)** $p4_3Ta_2$ (pink) with Δ6 increase from 50 V to 200 V for $[M+19H]^{19+}$. **(C)** CIU fingerprint of PFE and **(D)** $p4_3Ta_2$ with Δ6 increase from 50 V to 200 V for $[M+19H]^{19+}$

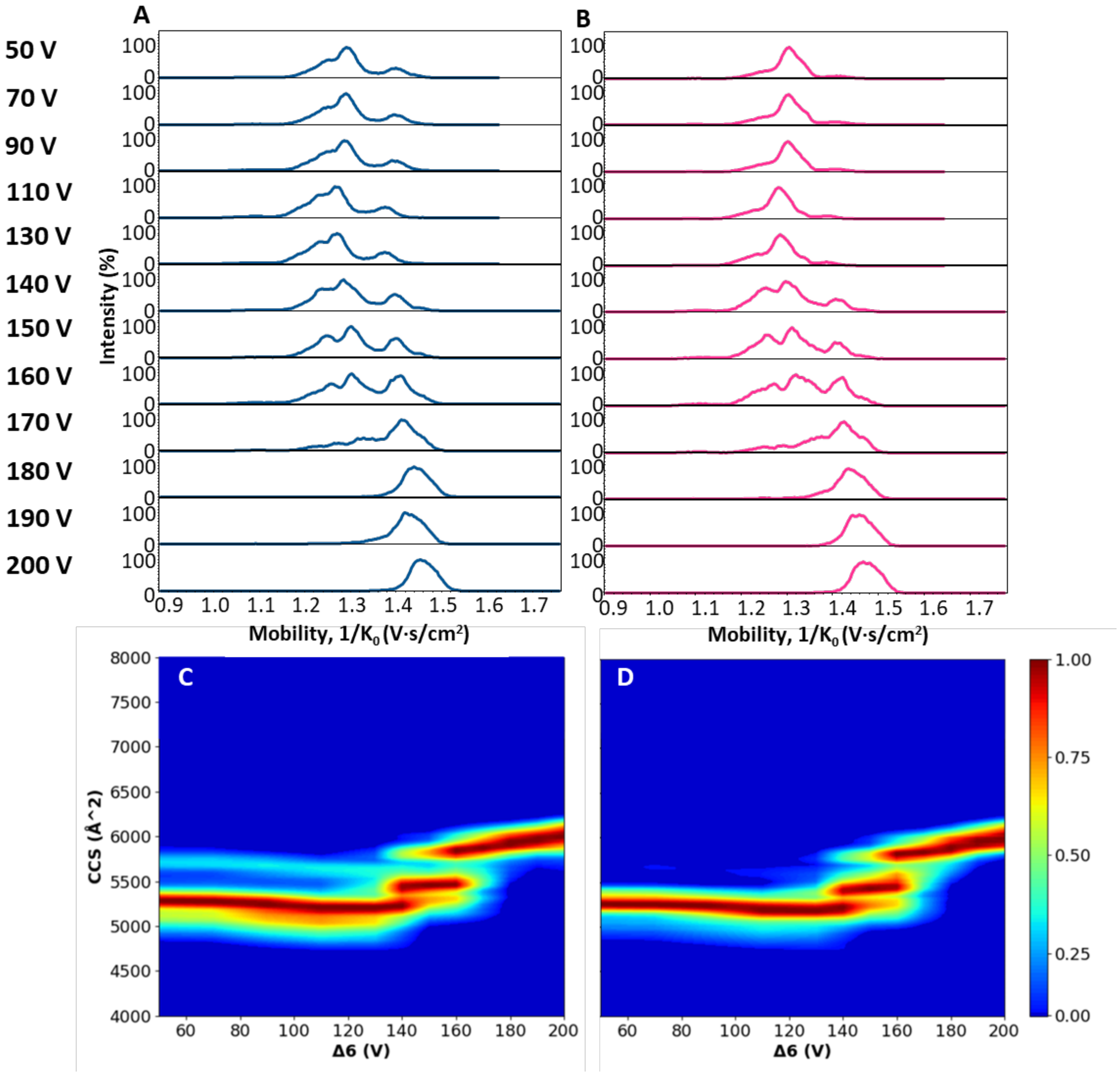

Figure S3.2 (A) Extracted mobility spectra of PFE (blue) **(B)** $p4_3Ta_2$ (pink) with Δ6 increase from 50 V to 200 V for $[M+20H]^{20+}$. **(C)** CIU fingerprint of PFE and **(D)** $p4_3Ta_2$ with Δ6 increase from 50 V to 200 V for $[M+20H]^{20+}$

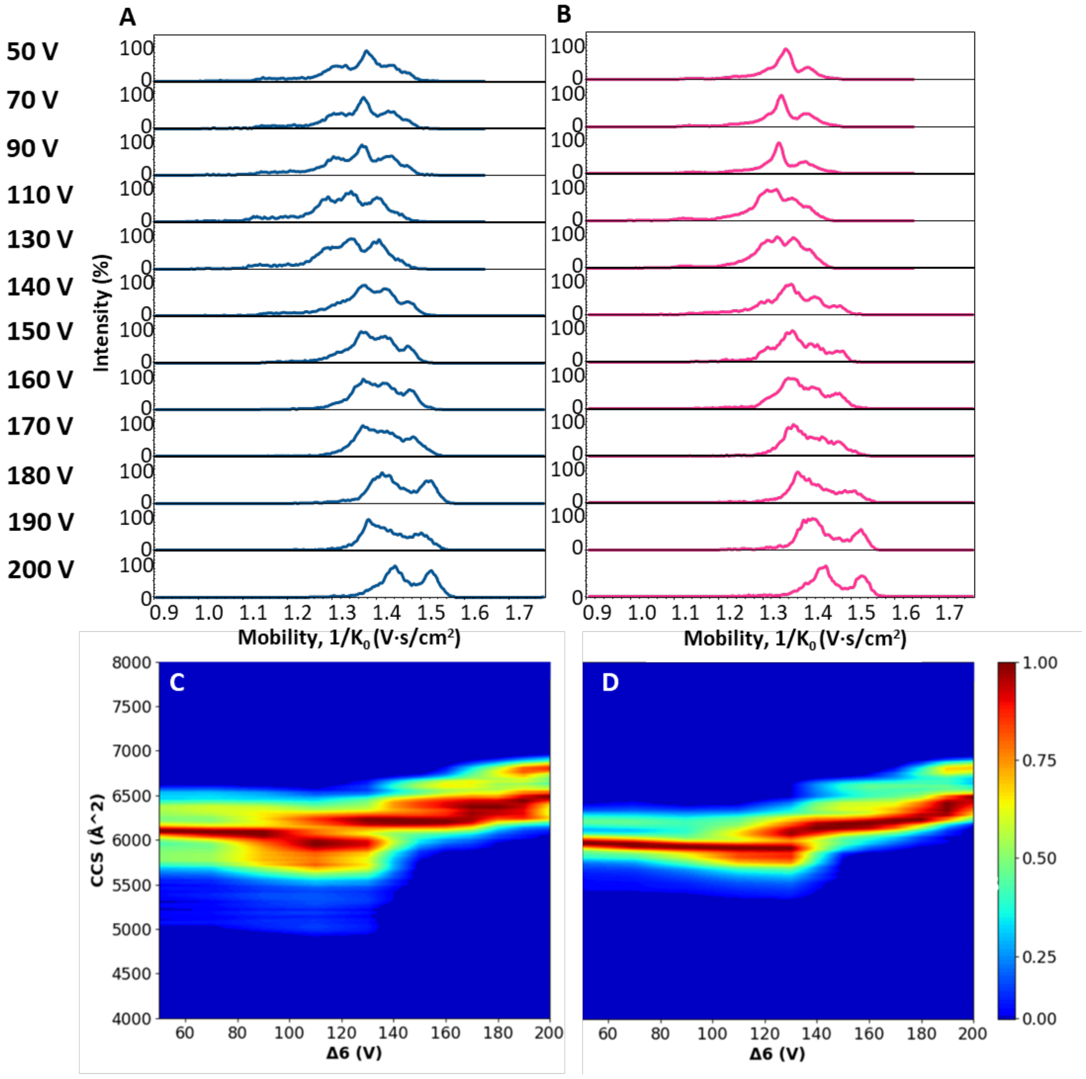

Figure S3.3 (A) Extracted mobility spectra of PFE (blue) **(B)** $p4_3Ta_2$ (pink) with Δ6 increase from 50 V to 200 V for $[M+22H]^{22+}$. **(C)** CIU fingerprint of PFE and **(D)** $p4_3Ta_2$ with Δ6 increase from 50 V to 200 V for $[M+22H]^{22+}$

Figure S4.1 to S4.4: CIU fingerprints of PFE and $p4_3Ta_2$ for $[M+19H]^{19+}$, $[M+20H]^{20+}$, $[M+21H]^{21+}$, $[M+22H]^{22+}$ measured at the tunnel in pressure of 1.7 mbar, 2 mbar, 2.2 mbar, and 2.6 mbar when increasing Δ6 from 50 V to 200V.

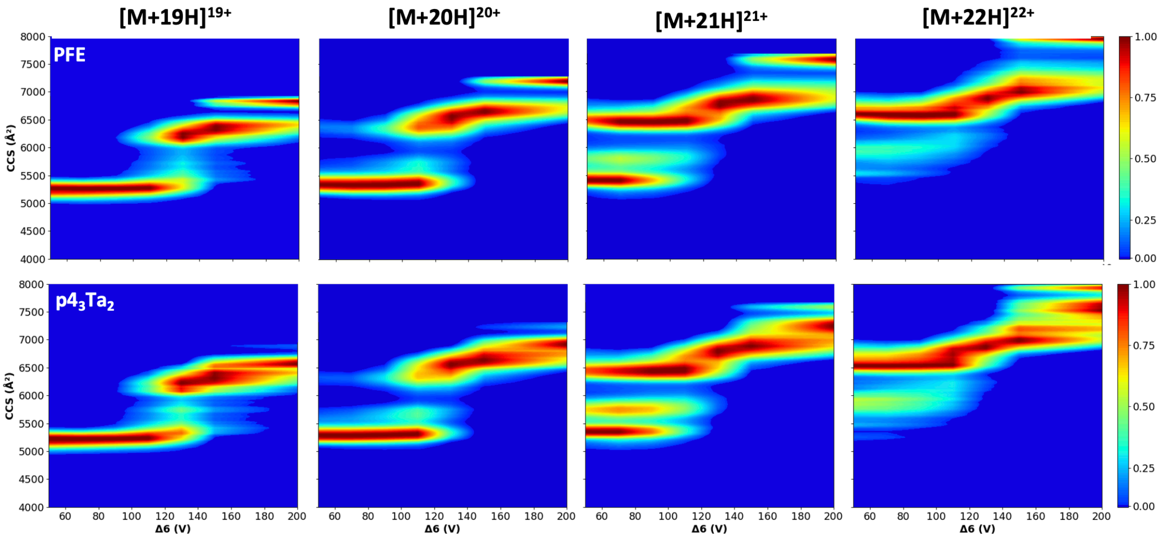

Figure S4.1. CIU fingerprint of PFE and $p4_3Ta_2$ measured at tunnel in pressure of 1.7 mbar with Δ6 increase from 50 V to 200 V for $[M+19H]^{19+}$, $[M+20H]^{20+}$, $[M+21H]^{21+}$, $[M+22H]^{22+}$.

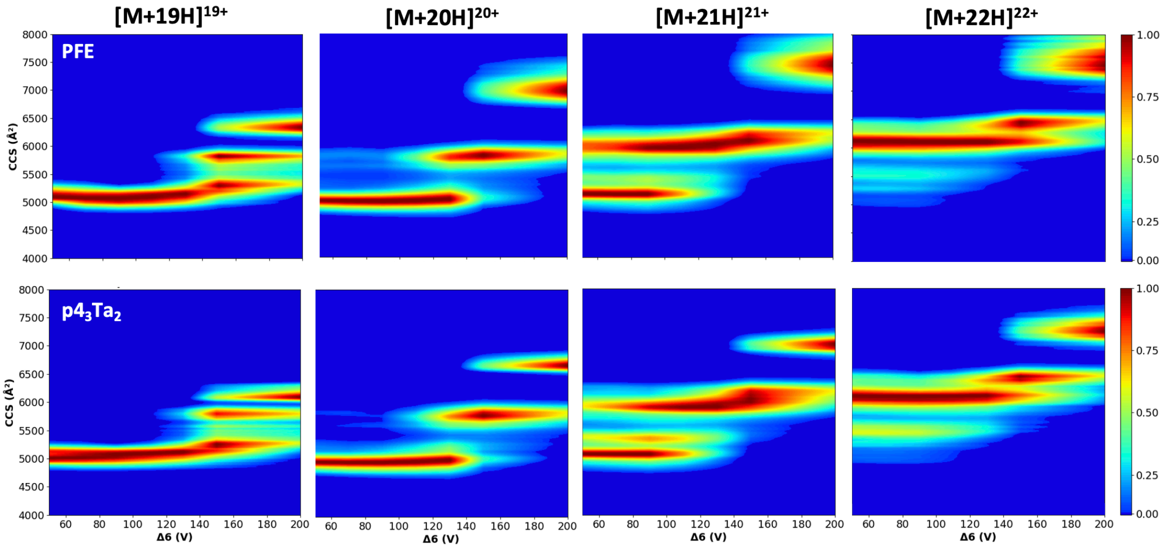

Figure S4.2. CIU fingerprint of PFE and $p4_3Ta_2$ measured at tunnel in pressure of 2.0 mbar with Δ6 increase from 50 V to 200 V for $[M+19H]^{19+}$, $[M+20H]^{20+}$, $[M+21H]^{21+}$, $[M+22H]^{22+}$.

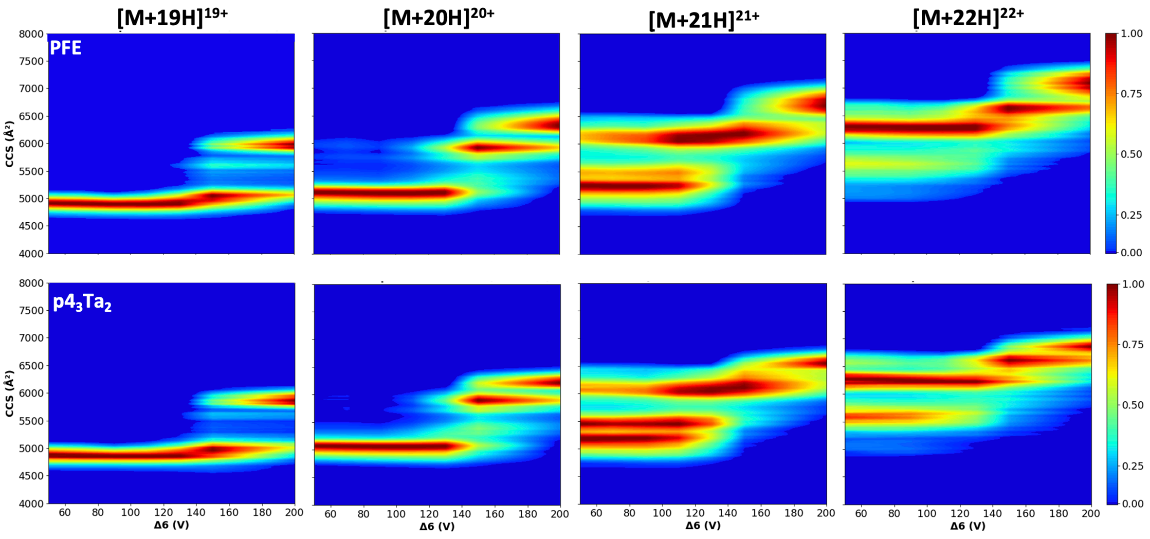

Figure S4.3. CIU fingerprint of PFE and p4$_3$Ta$_2$ measured at tunnel in pressure of 2.2 mbar with Δ6 increase from 50 V to 200 V for [M+19H]$^{19+}$, [M+20H]$^{20+}$, [M+21H]$^{21+}$, [M+22H]$^{22+}$.

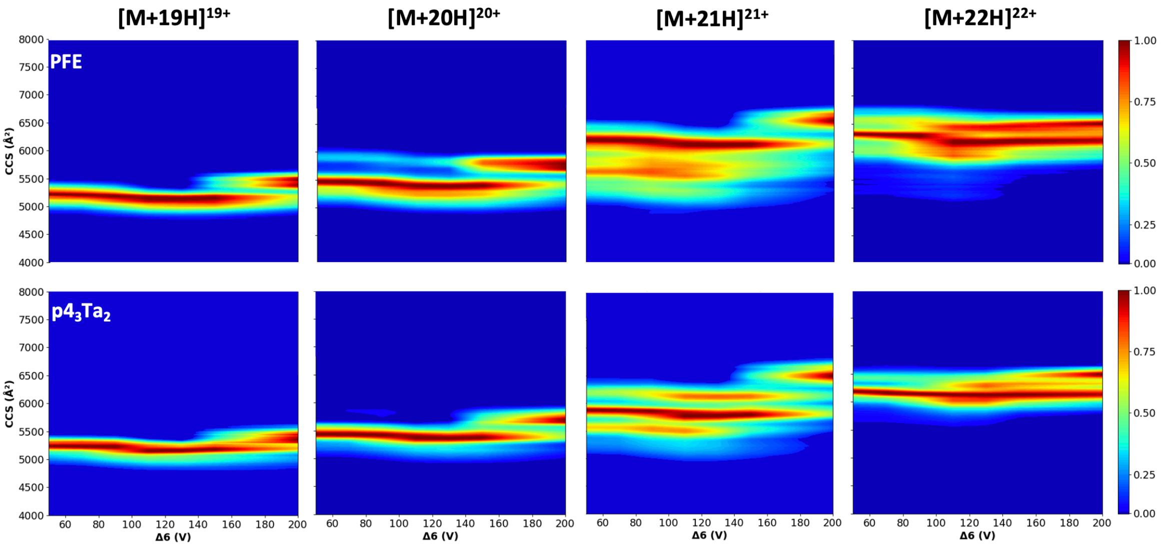

Figure S4.4. CIU fingerprint of PFE and p4$_3$Ta$_2$ measured at tunnel in pressure of 2.6 mbar with Δ6 increase from 50 V to 200 V for [M+19H]$^{19+}$, [M+20H]$^{20+}$, [M+21H]$^{21+}$, [M+22H]$^{22+}$.

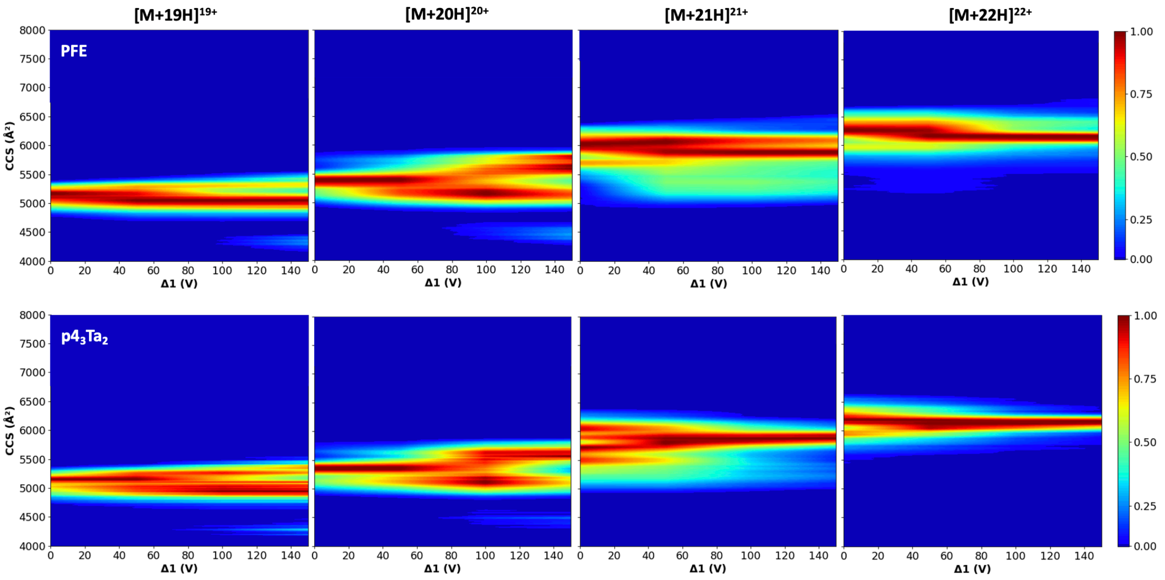

Figure S5. CIU fingerprint of PFE and p4$_3$Ta$_2$ measured at tunnel in pressure of 2.6 mbar with Δ1 increase from 0 V to 150 V for $[M+19H]^{19+}$, $[M+20H]^{20+}$, $[M+21H]^{21+}$, $[M+22H]^{22+}$.

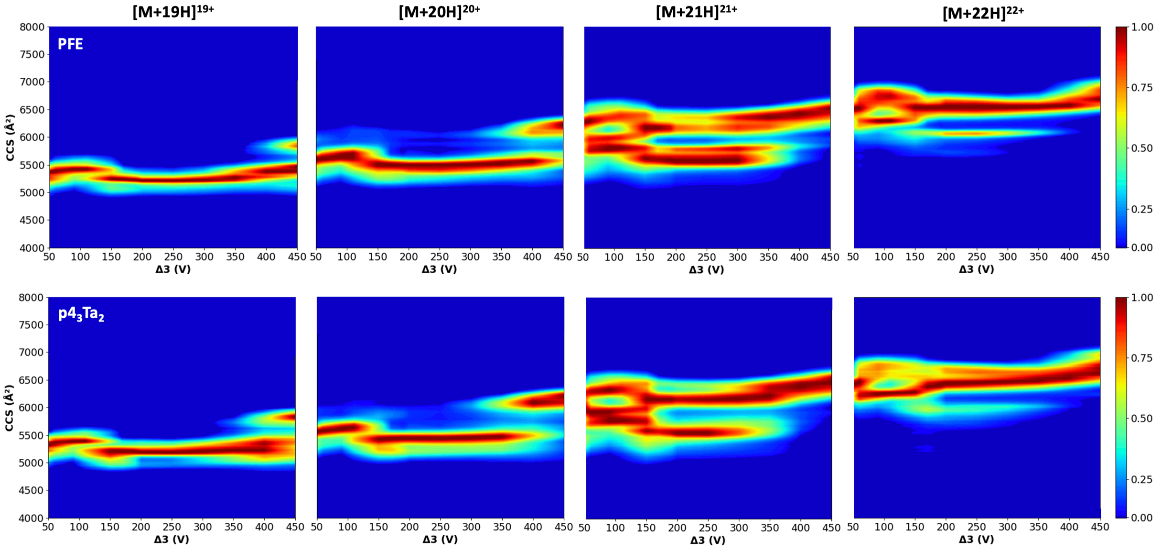

Figure S6. CIU fingerprint of PFE and p4$_3$Ta$_2$ measured at tunnel in pressure of 2.6 mbar with Δ3 increase from 50 V to 450 V for $[M+19H]^{19+}$, $[M+20H]^{20+}$, $[M+21H]^{21+}$, $[M+22H]^{22+}$.

Figure S7.1 to S.7.2: extracted mobility spectra of PFE and $p4_3Ta_2$ for $[M+19H]^{19+}$, $[M+20H]^{20+}$, $[M+21H]^{21+}$, $[M+22H]^{22+}$ measured with Δ3 voltages of 110 V, 150 V, and 170 V when increasing Δ6 from 70V to 200 V and Δ1 at 150 V and 200 V.

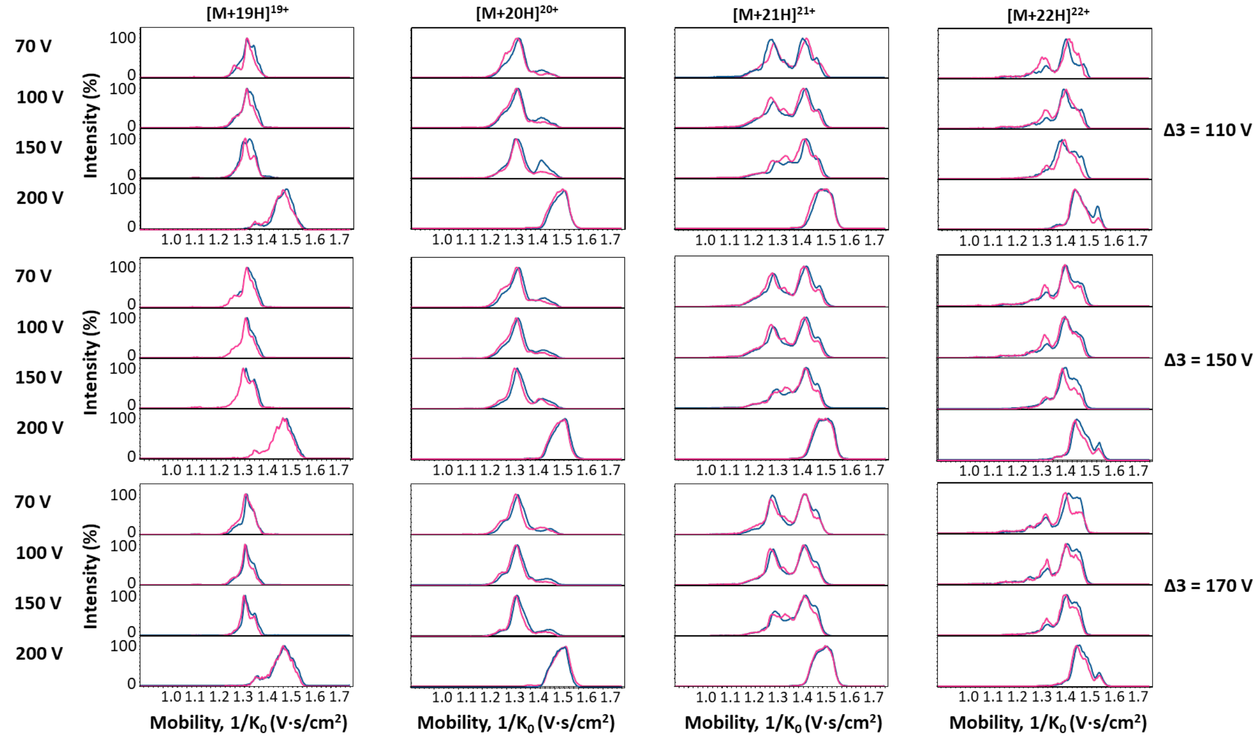

Figure S7.1. Extracted mobility spectra of PFE (blue) and $p4_3Ta_2$ (pink) measured at three Δ3 activation points of 110 V, 150 V, and 170 V while increasing the Δ6 from 70 V to 200 V for $[M+19H]^{19+}$, $[M+20H]^{20+}$, $[M+21H]^{21+}$, $[M+22H]^{22+}$.

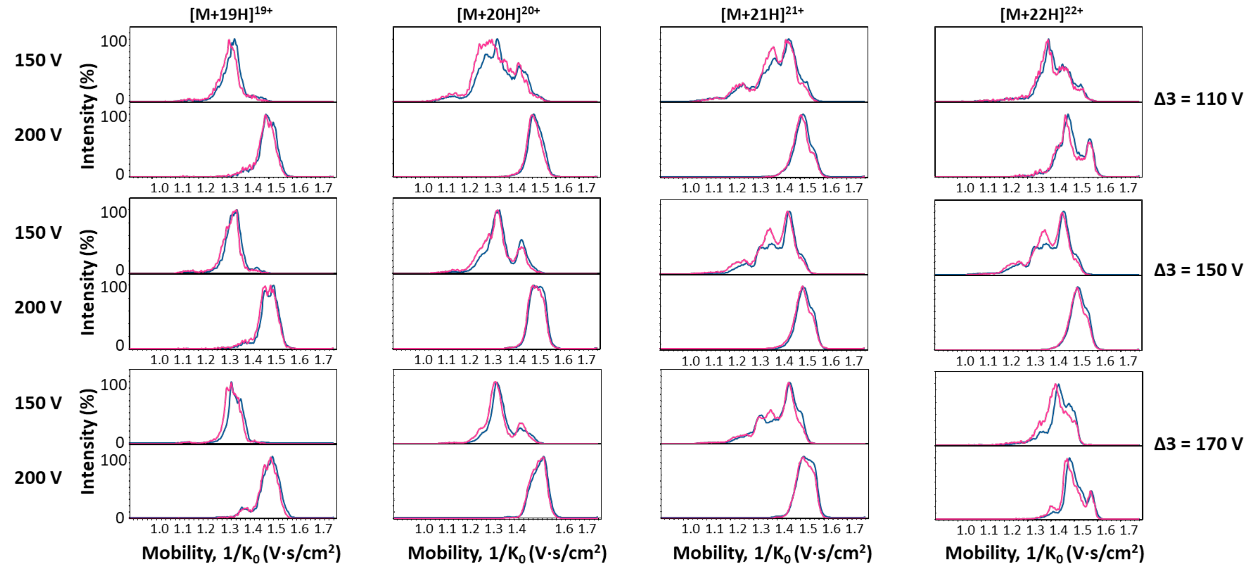

Figure S7.2. Extracted mobility spectra of PFE (blue) and $p4_3Ta_2$ (pink) measured at three Δ3 activation points of 110 V, 150 V, and 170 V while increasing the Δ1 from 50V to 200 V for $[M+19H]^{19+}$, $[M+20H]^{20+}$, $[M+21H]^{21+}$, $[M+22H]^{22+}$.

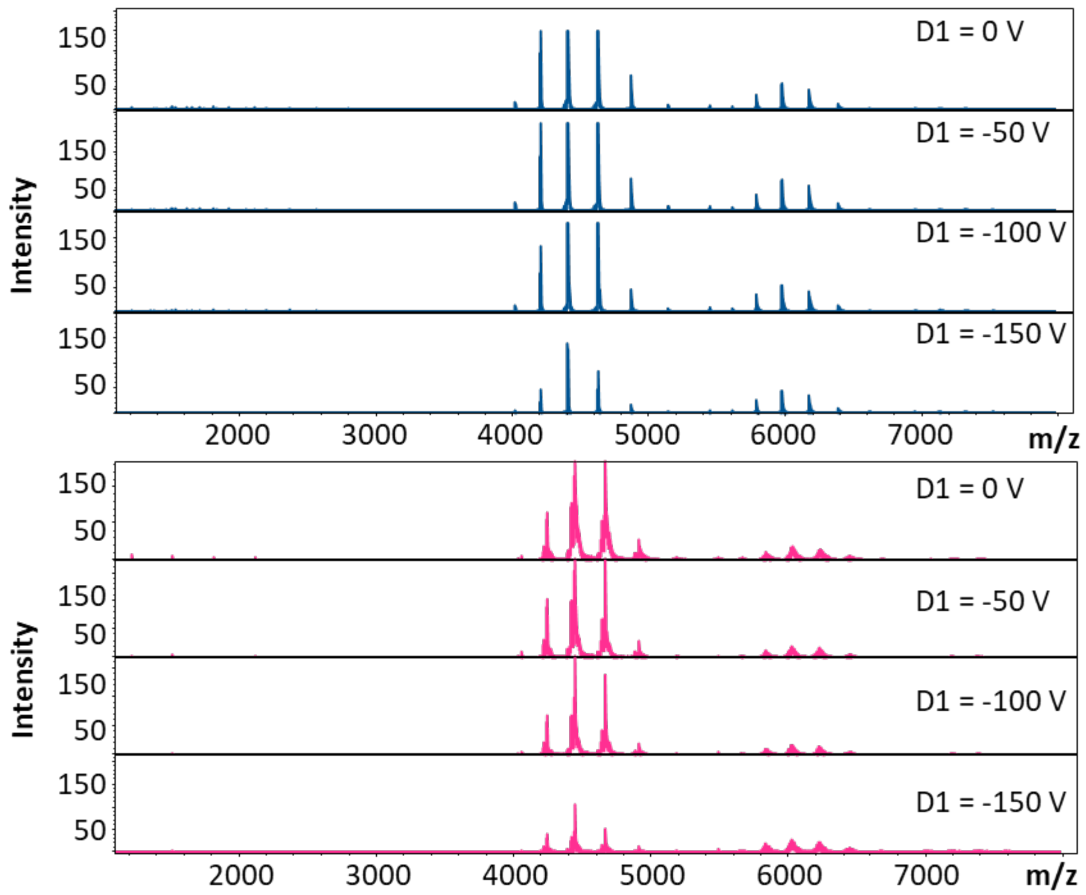

Figure S8. Total ion current (TIC) of PFE (blue) and $p4_3Ta_2$ (pink) measured while increasing the Δ1 from 0V to -150 V.

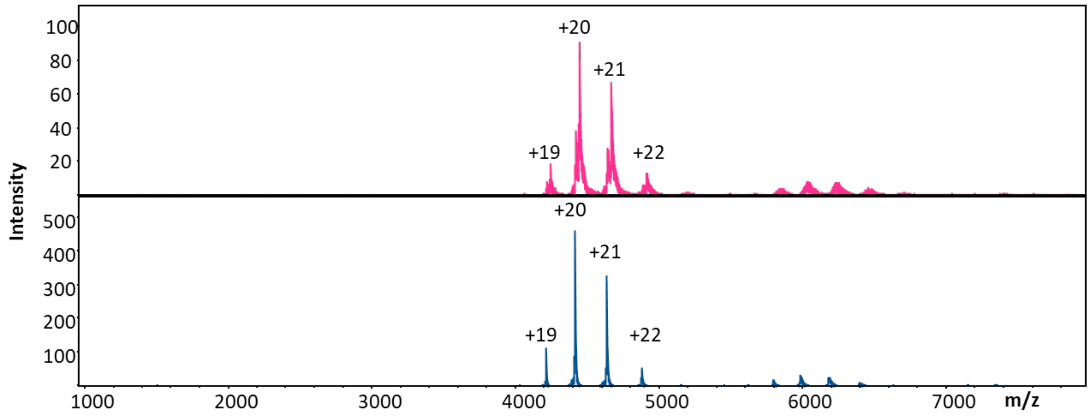

Figure S9. Total ion current (TIC) of PFE (blue) and $p4_3Ta_2$ (pink) measured at Δ6 voltages of 200V (max by the software)
